# A Chirality‐Converted Bacteriolytic Dodecapeptide Regulates *Vibrio*‐Induced Polymicrobial Infection and Ameliorates Invasion‐Associated Gut Microbiota Disequilibrium

**DOI:** 10.1002/advs.202522326

**Published:** 2026-04-16

**Authors:** Ping Zeng, Qipeng Cheng, Xiaoxu Zhang, Honglan Wang, Jinghan Zhang, Xinyi Ding, Pengfei Zhang, Lanhua Yi, Kwok‐Yin Wong, Kin‐Fai Chan, Sheng Chen, Sharon Shui Yee Leung

**Affiliations:** ^1^ School of Pharmacy, Faculty of Medicine The Chinese University of Hong Kong Shatin Hong Kong; ^2^ Anhui Provincial Key Laboratory of Molecular Enzymology and Mechanism of Major Metabolic Diseases College of Life Sciences Anhui Normal University Wuhu Anhui China; ^3^ Anhui Provincial Engineering Research Centre For Molecular Detection and Diagnostics College of Life Sciences Anhui Normal University Wuhu Anhui China; ^4^ School of Pharmacy Fudan University Shanghai China; ^5^ College of Food Science Southwest University Chongqing China; ^6^ State Key Laboratory of Chemical Biology and Drug Discovery and Department of Applied Biology and Chemical Technology The Hong Kong Polytechnic University Hung Hom Kowloon Hong Kong; ^7^ Department of Food Science and Nutrition, Faculty of Science The Hong Kong Polytechnic University Hung Hom Kowloon Hong Kong

**Keywords:** antibiofilm, cationic amphiphile, dextrorotatory amino acid, motility inhibition, mouse fecal microbiota, phage shock protein

## Abstract

The polymicrobial infections caused by *Vibrio* species, especially multidrug‐resistant isolates, are posing increasing threats to the health of coastal residents. Herein, a novel dodecapeptide, denoted as **D‐zp37**, was designed and synthesized, aiming to combat these notorious pathogens. Experimental results proved that the simple chirality conversion of the parent peptide, **zp37**, could significantly boost the antibacterial activity and proteolytic stability. To a specific cephalosporin‐resistant *Vibrio alginolyticus*, the minimal inhibitory concentration value of **D‐zp37** was as low as 0.5 µm. Beyond the strong bacteriolytic effect against planktonic *Vibrio alginolyticus*, *Vibrio parahaemolyticus* and *Vibrio vulnificus* strains, **D‐zp37** was found to restrain the biofilm establishment of *Vibrio* mixtures via inhibiting the efflux of intracellular polysaccharides. Mechanistic studies hinted that **D‐zp37** impeded the electron transport chain, impaired the membrane stress response by downregulating the phage shock protein family, and blocked amino acid biosynthesis in *Vibrio* cells. Furthermore, three different infection models (shrimp, *Galleria mellonella* larvae and immunosuppressed mice) collaboratively confirmed the anti‐*Vibrio* efficacy of **D‐zp37** in vivo. In addition, the intragastric administration of **D‐zp37** was found to be beneficial in restoring the imbalanced gut microbiota induced by the polymicrobial *Vibrio* invasion. In summary, this work investigated the antibacterial mechanisms and in vivo efficacy of a novel peptide lead compound, proposing a new therapeutic against *Vibrio* infections.

## Graphical abstract

Multidrug‐resistant *Vibrio* infections are rising rapidly and threaten coastal populations worldwide. This study introduces **D‐zp37**, a chirality‐engineered antimicrobial peptide with exceptional potency against resistant *Vibrio* species. **D‐zp37** kills planktonic cells, blocks mixed‐species biofilms, disrupts essential bacterial stress responses, and shows strong therapeutic performance in shrimp, larvae, and mouse infection models. These results position **D‐zp37** as a promising new therapeutic candidate for combating dangerous *Vibrio* infections.

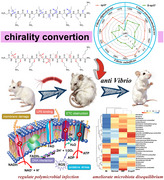

## 1 Introduction

*Vibrio* is a genus of Gram‐negative (G‒ve) bacteria widely distributed in coastal waters and inside bodies of marine animals and many of them are aquatic pathogens [1]. *Vibrio* infections can lead to production loss of seafood and threaten the safety of final consumers. For human beings, *Vibrio* invasion can be acquired via intaking contaminated seafood or exposing open wounds to bacteria‐rich environments. The resulting vibrioses, represented by necrotizing fasciitis and watery diarrhea, pose potential health threats, especially to immunocompromised coastal residents [2]. Although great efforts have been put into fighting against *Vibrio* infections, hitherto they remain a thorny issue [3]. It is predicted that the population at risk of *Vibrio* infections would reach up to 1.3 billion by 2050 [4].

Clinically, commonly used antibiotics against *Vibrio* species include tetracycline, gentamicin, fluoroquinolones and *β*‐lactam antibiotics [5]. However, the abuse of these medications and the dissemination of plasmid‐mediated resistance greatly impair their effectiveness in controlling *Vibrio* infections [6]. Isolation of multidrug‐resistant (MDR) *Vibrio alginolyticus* (*VA*), *Vibrio parahaemolyticus* (*VP*) and *Vibrio vulnificus* (*VV*) were frequently reported [7]. Occurrence of tetracycline resistance gene *tet*(M) was first detected in *Vibrio* species about 20 years ago, while the novel metallo‐β‐lactamase VAM‐1, which conferred reduced carbapenem susceptibility, was identified more recently, highlighting the evolving antimicrobial resistance in this genus [8]. The constant warming marine environment is also believed to intensify the global expansion of MDR *Vibrio* [9].

Mature biofilms formed by *Vibrio* species can persist on the surfaces of commonly consumed seafoods [10]. This dense and complex extracellular matrix impedes the interaction between embedded bacterial cells and antibacterial agents, thus helping pathogens escape external antibacterial attacks [11]. Furthermore, in real‐world environments, diverse microorganisms can build cooperative communities to promote co‐existence [12]. Their spatial organization within wound sites can result in refractory polymicrobial infections [13].

Antimicrobial peptides (AMPs), originated from the host defense system, are considered promising alternatives in combating pathogens, especially in this antimicrobial resistance era. [14] The outer membrane (OM) of G‒ve *Vibrio* species presents a significant permeability barrier to many small‐molecule antibiotics [15]. In the meantime, the large amount of negatively charged lipopolysaccharide (LPS) on the OM surface could serve as important targets for cationic AMPs through electrostatic interactions. Therefore, designing LPS‐targeting peptides becomes a simple yet effective strategy for targeting G‒ve bacteria [16].

Previously, we identified a peptide **zp3** (GIIAGIIIKIKK‐NH_2_) from a fragment of the *Staphylococcus aureus* toxin phenol‐soluble modulin, which showed antibacterial activity against both *Escherichia coli* and *Bacillus subtilis* [17]. Through targeted amino acid substitutions, its analog **zp37** (GIKAKIIIKIKK‐NH_2_) exhibited an expanded antibacterial spectrum and improved bioactivity [18]. However, the sequence of **zp37** consists entirely of proteinogenic amino acids, resulting in poor proteolytic stability, which significantly limits its clinical potential. In this study, 11 residues of **zp37**, except the capping glycine from the *N*‐terminal, were replaced with their corresponding dextrorotatory chiral isomers. This novel compound, denoted as **D‐zp37**, was investigated for its antibacterial capability against polymicrobial *Vibrio* infections using both in vitro and in vivo models.

## 2 Results

### 2.1 Chirality Conversion Enhances In Vitro Stability and Antibacterial Potential Against MDR *Vibrio* Strains of Designed Dodecapeptide

**FIGURE 1:**
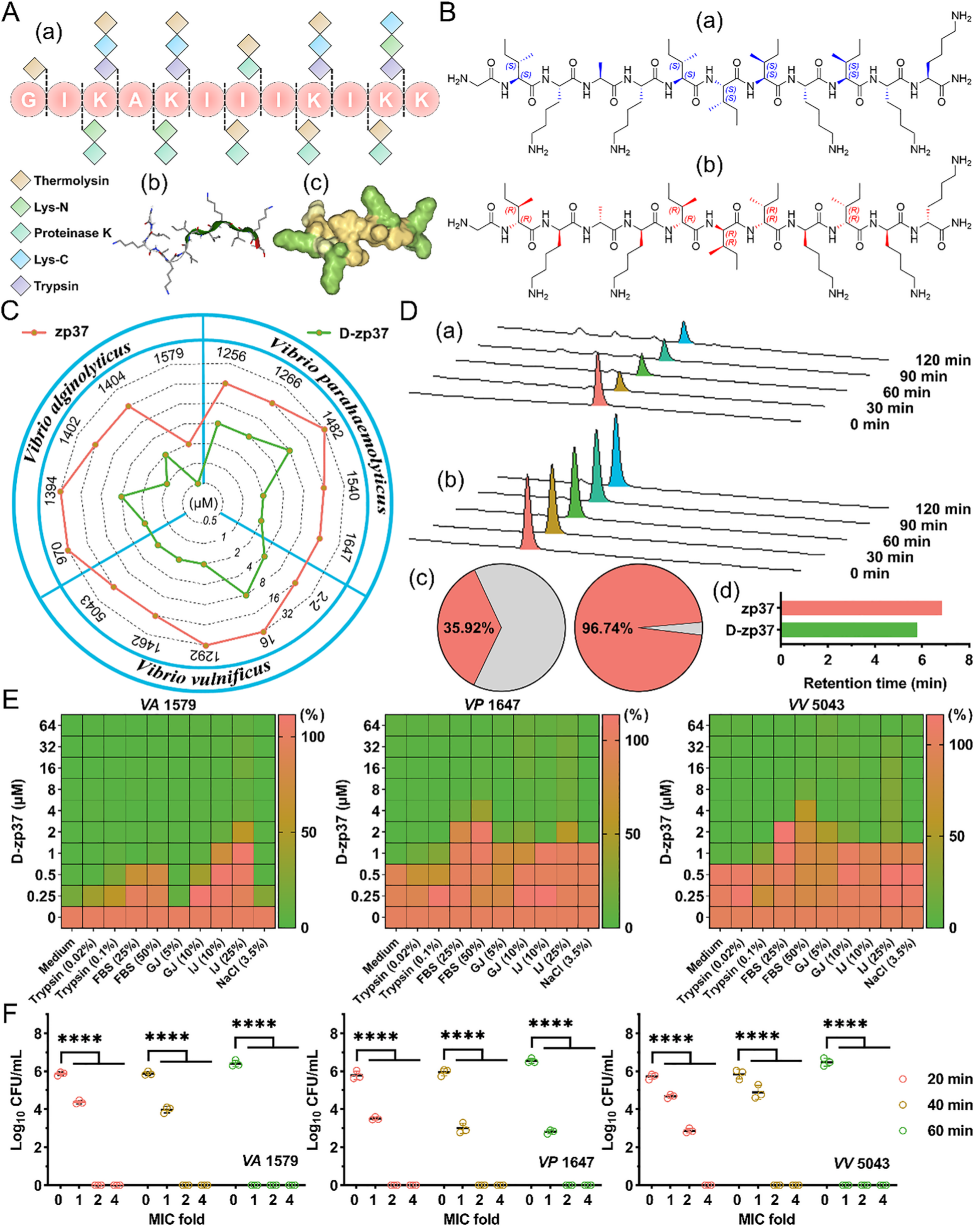
(A) In silico predicted possible protease cleavage sites (a) of **zp37**. Ribbon diagram (b) indicates secondary structure and surface diagram (c) indicates Van der Waals surface based on hydrophobicity. (B) Chemical structures of **zp37** (a) and **D‐zp37** (b). (C) MIC values of **zp37** and **D‐zp37** against *Vibrio* strains. (D) HPLC diagrams of **zp37** (a) and **D‐zp37** (b) after incubating with 10 µg/mL trypsin. Remaining peak areas (c) of **zp37** and **D‐zp37** after 120 min treatment. Retention time (d) of **zp37** and **D‐zp37**. (E) Bacterial growth in various conditions in the presence of different concentrations of **D‐zp37**. (F) Live bacterial counting after **D‐zp37** treatment.

Peptide **zp37** was predicted to be sensitive to many common enzymes, such as thermolysin, proteinase K, and trypsin. Possible protease cleavage sites were visualized (Figure 1A(a)). Its helical structure and amphiphilic interface were shown in Figure 1A(b,c) respectively. To improve the proteolytic stability of **zp37**, its chirality‐converted analogue was engineered by replacing all L‐isoleucine (*2S*, *3S*) with D‐isoleucine (*2R*, *3R*). This novel peptide was denoted as **D‐zp37**. Chemical structures of **zp37** and **D‐zp37** were shown in Figure 1B(a,b), respectively.

We first evaluated the antibacterial activity of the original and modified peptides against *Vibrio* bacteria using a double‐dilution assay. For the 15 tested MDR *Vibrio* isolates (5 *VA*, 5 *VP* and 5 *VV* strains), the minimal inhibitory concentration (MIC) values of **D‐zp37** were at least 4‐fold lower than that of the parent peptide, **zp37**, and in the range of 0.5 to 8 µm (Figure 1C).

The primary objective of engineering **zp37** to **D‐zp37** is to improve the proteolytic stability. We therefore compared their trypsin tolerance using high performance liquid chromatography (HPLC) to quantify the rate of degradation. Upon the change of chirality of isoleucine in the peptide sequence, the retention time of **zp37** (6.8 min) was shifted to 5.8 min for **D‐zp37** (Figure 1D(d)), which was common among chiral enantiomers [19]. The chromatography results revealed that **zp37** degraded significantly within 30 min in the presence of 10 µg/mL trypsin (Figure 1D(a)). Differently, the same concentration of trypsin would hardly cause degradation of **D‐zp37** throughout the entire monitoring period (Figure 1D(b)). After 120 min incubation, the remaining peak areas of **zp37** and **D‐zp37** were 35.92% and 96.74%, respectively (Figure 1D(c)).

To further evaluate the environmental tolerance of **D‐zp37**, its bacterial inhibition effect was measured in various harsh conditions. We selected one strain from each of the three *Vibrio* species as indicators, namely *VA* 1579, *VP* 1647 and *VV* 5043. Figure 1E depicted the level of bacterial growth after incubating in normal medium, trypsin, fetal bovine serum (FBS), gastric juice (GJ), intestinal juice (IJ), and 3.5% NaCl solution in the presence of different concentrations of **D‐zp37** for 6 h. All three *Vibrio* cells can be at least partially inhibited in the presence of 2 µm **D‐zp37** in all tested scenarios.

Figure 1F revealed that 20 min treatment with **D‐zp37** at the respective MIC‐level was able to significantly reduce the colony forming unit (CFU) for all *Vibrio* species. Moreover, **D‐zp37** at 2‐fold MICs could eradicate *VA* 1579 and *VP* 1647 to undetectable levels in 20 min.

### 2.2 D‐zp37 Demonstrates Selectively Bacteriolytic Effect Against *Vibrio* Cells

**FIGURE 2:**
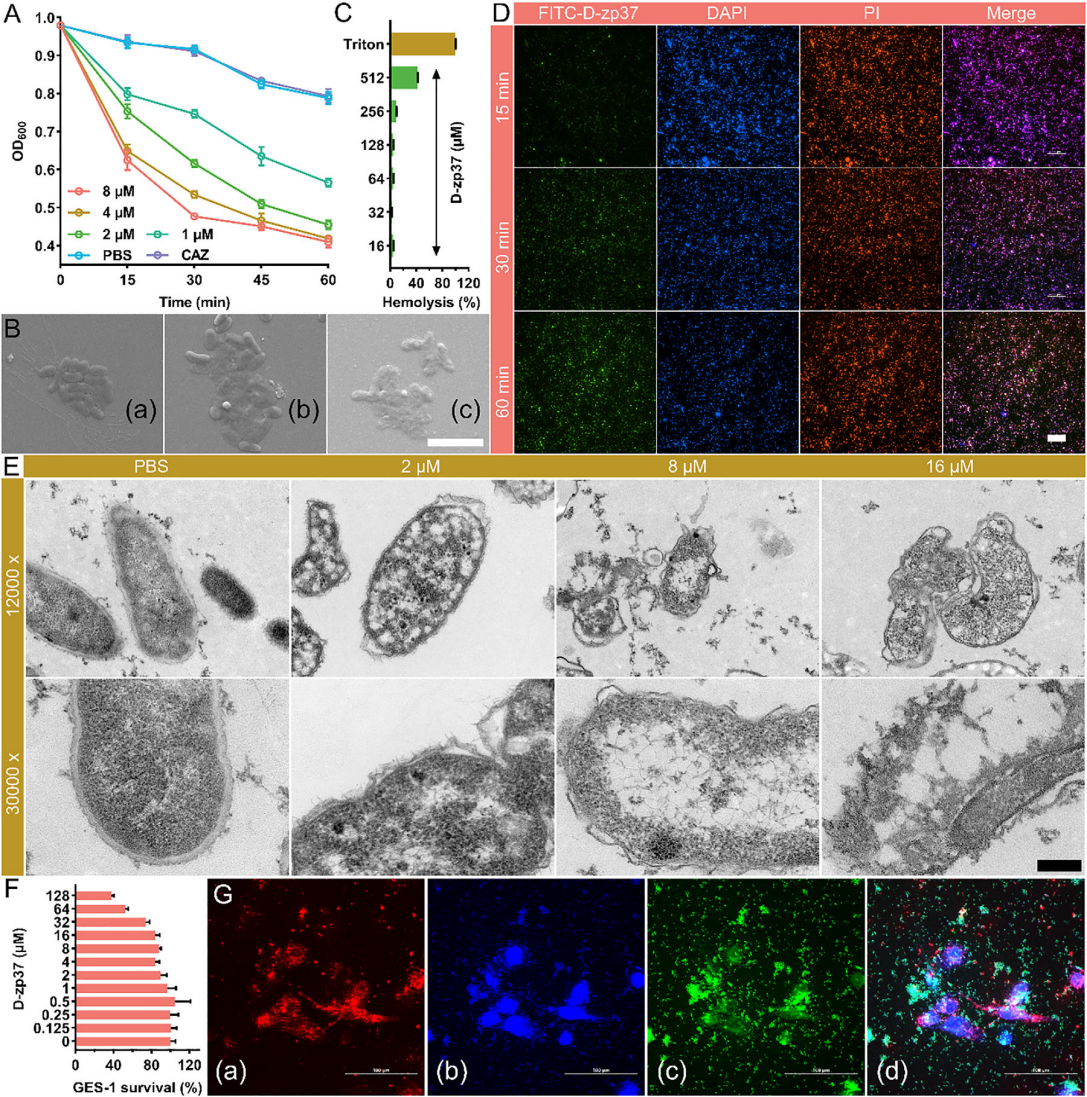
(A) Turbidity changes of *Vibrio* mixture after **D‐zp37** or 8 µm CAZ treatment. (B) SEM images of *Vibrio* mixture after PBS (a), 2 µm **D‐zp37** and 8 µm **D‐zp37** treatment. Scale bar: 5 µm. (C) Hemolytic activity of **D‐zp37** against RBCs. (D) Triple fluorescence staining of *Vibrio* mixture by **FITC‐D‐zp37**, DAPI and PI. Scale bar: 100 µm. (E) TEM images of the *Vibrio* mixture. Scale bar: 200 nm. (F) Cytotoxicity of **D‐zp37** against GES‐1 cells. (G) Co‐culture system of *Vibrio* mixture and GES‐1 cells stained by Nile red, Hoechst 33258 and **FITC‐D‐zp37**. Scale bar: 100 µm.

Co‐existence of various pathogens gives rise to polymicrobial infection [20]. The isolation of multiple *Vibrio* species from the same aquatic animal sample as primary colonizers has also been reported [21]. Therefore, a bacterial mixture composed of an equivalent amount of *VA* 1579, *VP* 1647 and *VV* 5043 was set as a model to evaluate the efficacy of **D‐zp37** against *Vibrio* polymicrobial infection. First, the turbidity assay suggested that **D‐zp37** exhibited bacteriolytic effect on the *Vibrio* mixture (Figure 2A). The optical density at 600 nm (OD_600_) values dropped significantly after 15 min peptide treatment, indicating that **D‐zp37** was able to lyse the *Vibrio* mixture rapidly. Comparatively, antibiotics ceftazidime (CAZ) exhibited no killing effect at the tested concentration. Scanning electron microscopy (SEM) images described a portrait of *Vibrio* cells treated with phosphate buffered saline (PBS), 2 or 8 µm **D‐zp37** respectively (Figure 2B). The morphology of bacterial cells became collapsed and fused after the peptide treatment. The loss of a well‐defined boundary clarified that *Vibrio* cells were being lysed by **D‐zp37**. While for red blood cells (RGB), **D‐zp37** showed a negligible hemolytic effect even at 256 µm (Figure 2C).

We further investigated the intracellular penetration kinetics of the peptide using fluorescein isothiocyanate‐labelled **D‐zp37** (**FITC‐D‐zp37**) and fluorescence microscopy (Figure 2D). According to the images of the first column, the green fluorescence emitted from *Vibrio* cells could be observed in 15 min. With time elapsing, the fluorescence intensity increased significantly, suggesting that a growing amount of **D‐zp37** molecules had crossed the cell membrane. On the contrary, blue fluorescence from *4’,6*‐diamidino‐*2*‐phenylindole (DAPI), a DNA specific probe, gradually weakened, which hinted that DNA leakage may happen during bacterial lysis. Propidium iodide (PI) staining reconfirmed the bactericidal effect of **D‐zp37**.

Fine structural changes of *Vibrio* after being attacked by **D‐zp37** were captured by transmission electron microscopy (TEM) (Figure 2E). Bacterial cells in the PBS‐treated control group presented a healthy state of growth. In contrast, bacteria subjected to **D‐zp37** treatment exhibited anomalous morphology including membrane exfoliation (2 µm group), cell deformation and cytoplasm leakage (4 µm group), and severe structural distortion and even complete bacterial lysis (8 µm group).

The digestive tract is a high incidence site for *Vibrio* polymicrobial infection. To assess whether this molecule adversely affected normal host cells, we tested its cytotoxicity to the human gastric epithelial cell line GES‐1 (Figure 2F). Cell counting kit‐8 (CCK‐8) assay reported the half‐maximal inhibitory concentration (IC_50_) of **D‐zp37** against GES‐1 was >64 µm, much higher than the MICs against *Vibrio* bacteria. Moreover, when a *Vibrio* mixture was co‐cultured with GES‐1 cells, **D‐zp37** demonstrated a stronger affinity to bacterial cells. This was evidenced by fluorescence microscopy, where green fluorescence emitted by FITC‐D‐zp37 was predominantly observed within bacterial cells (stained in red) rather than mammalian cells (stained in blue) (Figure 2G).

### 2.3 D‐zp37 Restrains Bacterial Motility and Combats Biofilm Establishment via Inhibiting Efflux of Intracellular Polysaccharide

**FIGURE 3:**
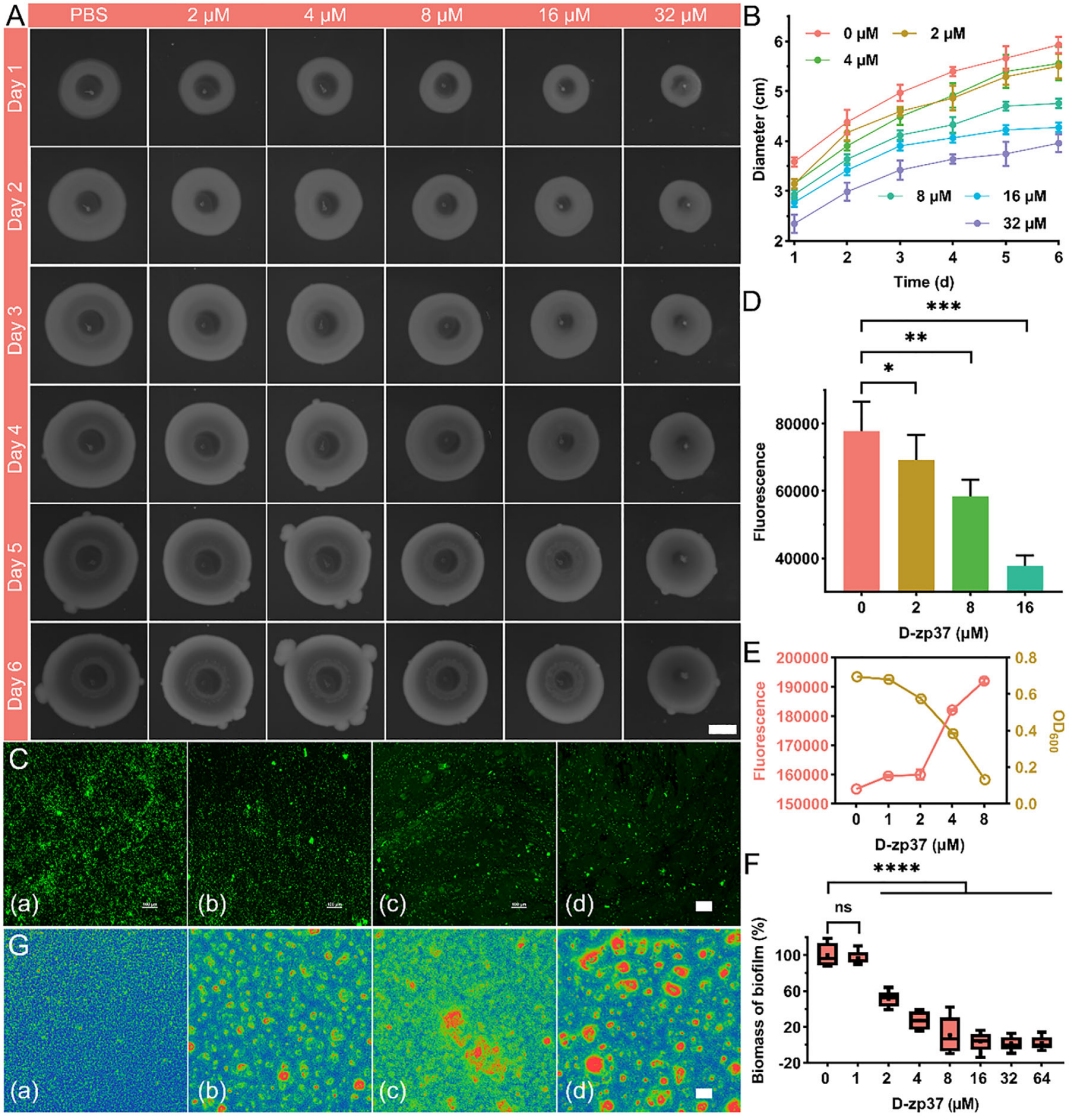
(A) Swimming behaviour of *Vibrio* mixture in agar plates containing different concentrations of **D‐zp37**. Scale bar: 2 cm. (B) Average swimming diameters of *Vibrio* mixture. (C) Fluorescence images of exopolysaccharide expressed by *Vibrio* mixture after PBS (a), or 2 µm (b), 8 µm (c), 16 µm (d) **D‐zp37** treatment. Scale bar: 100 µm. (D) Quantitative analysis of exopolysaccharide content before and after **D‐zp37** treatment. (E) Turbidity changes of *Vibrio* mixture and corresponding intracellular polysaccharide content before and after **D‐zp37** treatment. (F) Productive biomass of biofilm in the absence or presence of **D‐zp37**. (G) Phase images of biofilm treated with PBS (a), or 16 µm (b), 32 µm (c), 64 µm (d) **D‐zp37**. Scale bar: 100 µm.

The flagella of *Vibrio* bacteria could boost motility, thereby facilitating their fast spread [22]. Swimming behaviour, as one of the representative forms of motion, is often monitored to evaluate whether an antimicrobial agent can suppress cluster migration of bacterial cells. For six consecutive days, we recorded the swimming images of *Vibrio* mixture on the surface of an agar plate containing different concentrations of **D‐zp37** (Figure 3A). In general, the bacterial swimming area of ​​each group gradually expanded over time. However, in parallel comparison, the swimming area decreased with increasing **D‐zp37** concentration, suggesting that the peptide could inhibit the migration of *Vibrio* cells in a dose‐dependent manner. Average diameters of 0 µm and 32 µm groups shown in Figure 3B were 3.58 and 2.35 cm on Day 1. The difference between the two groups was 1.23 cm. While on Day 6, such value widened to 1.97 cm (5.93 and 3.96 cm respectively).

Producing biofilm is an effective strategy for bacteria to resist antibiotics, necessitating the antibiofilm capability of AMPs for effective control [23]. Though the compositions of biofilms are complex, exopolysaccharide (EPS) are considered to be the core component [24]. The secretion amount of EPS after the **D‐zp37** treatment was then visualized (Figure 3C) and quantitatively analyzed (Figure 3D). Fluorescence intensity of Alexa Fluor 488 conjugated concanavalin A decreased significantly when **D‐zp37** was applied, suggesting that *Vibrio* mixture released less EPS in the presence of **D‐zp37**. Interestingly, when we tested the intracellular polysaccharide of the *Vibrio* mixture, it can be found that its content continued to increase despite sharply dropping bacterial turbidity (Figure 3E). After normalization, intracellular polysaccharide contents per unit cell rose 1.05‐fold (1 µm), 1.25‐fold (2 µm), 2.13‐fold (4 µm) and 6.53‐fold (8 µm) compared with the untreated control, respectively.

As mentioned before, EPS play a key role in forming the biofilm matrix. The diminishment of EPS after **D‐zp37** treatment was also reflected in the decrease of biomass of the biofilm (Figure 3F). Treating *Vibrio* mixture with 2 µm **D‐zp37** could have a dramatical impact on biofilm establishment and bacterial cells incubated with >8 µm **D‐zp37** basically lost the ability to produce biofilm. Moreover, **D‐zp37** was able to dissipate pre‐formed biofilms (Figure 3G), Phase contrast images captured the difference of *Vibrio* biofilm treated with PBS, 16, 32 and 64 µm **D‐zp37**. Pre‐formed biofilm presented a dense and uniform structure (a of Figure 3G). However, **D‐zp37** molecules triggered inhomogeneity in a dose‐dependent manner (b–d of Figure 3G), indicating that parts of the biofilm were dispersed.

### 2.4 D‐zp37 Impedes Electron Transport Chain (ETC) and Triggers Abnormal Intracellular Reactive Oxygen Species (ROS) Accumulation

**FIGURE 4:**
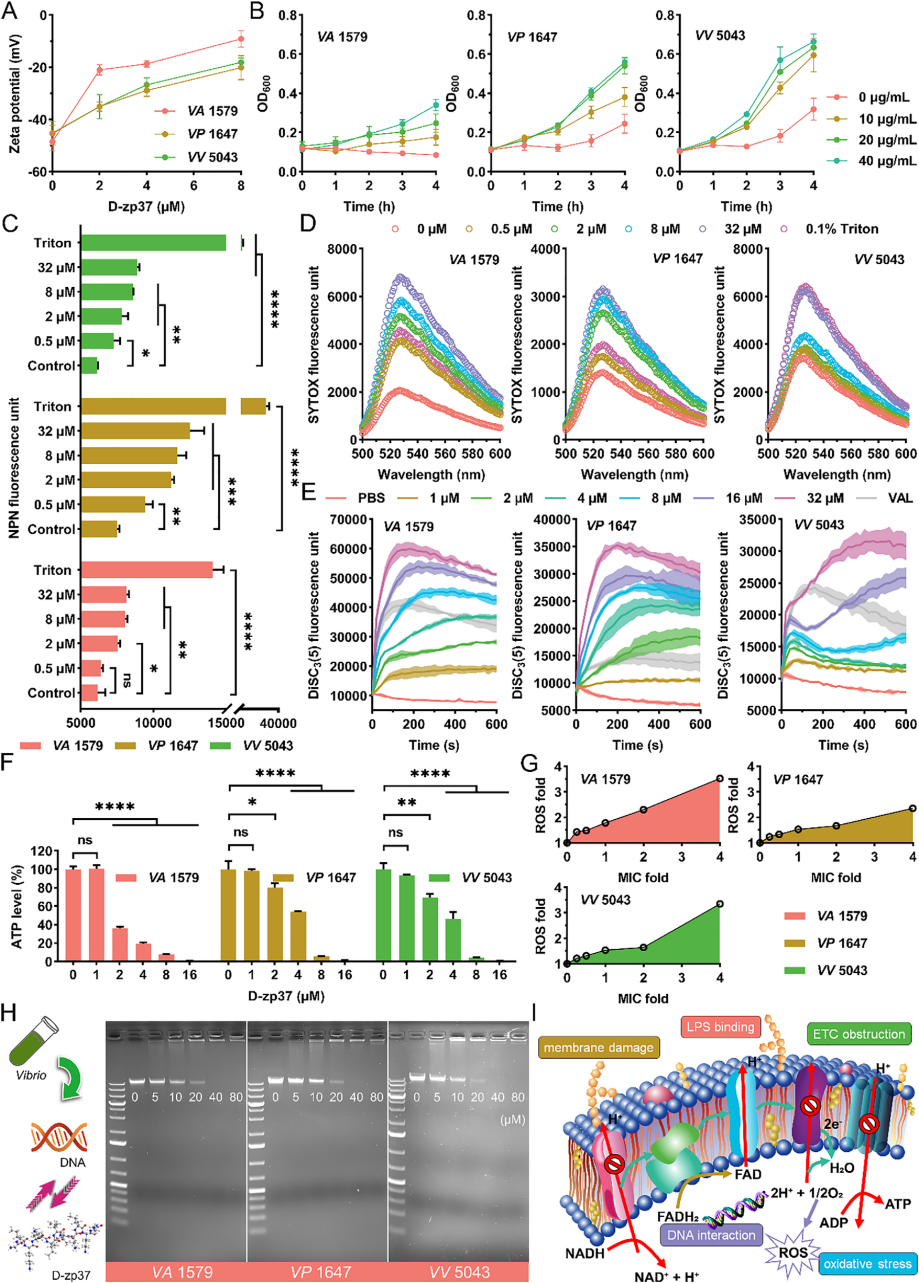
(A) Zeta potential change of *Vibrio* cells before and after **D‐zp37** treatment. (B) Growth kinetics of *Vibrio* cells in the presence of extra LPS. (C) OM permeability change of *Vibrio* cells. (D) IM permeability change of *Vibrio* cells. (E) Membrane potential change of *Vibrio* cells. (F) ATP level change of *Vibrio* cells. (G) ROS accumulation of *Vibrio* cells. (H) Interaction assessment between **D‐zp37** and *Vibrio* DNA by gel retardation analysis. (I) Schematic illustration of the antibacterial mode of **D‐zp37**.

We first monitored the change of zeta potential of *VA* 1579, *VP* 1647 and *VV* 5043 in the presence of **D‐zp37** (Figure 4A). The upward trends from approximately −50 to −10 mV (*VA* 1579) or around −20 mV (*VP* 1647, *VV* 5043) with increasing concentration of **D‐zp37** suggested that this peptide could interact with LPS to neutralize the membrane potential. To verify this hypothesis, LPS of *VA* 1579, *VP* 1647 and *VV* 5043 were first isolated and added to cultures containing fresh bacteria and 5 µm **D‐zp37**. We found that a higher concentration of exogenous LPS could effectively suppress the antibacterial activity of **D‐zp37**, resulting in better bacterial growth (Figure 4B). In addition, from the isothermal titration calorimetry (ITC) data, the calculated dissociation constant (K_d_) between **D‐zp37** and LPS was 4.8 ± 24.1 nm (Figure S1).

The outer membrane (OM) and inner membrane (IM) permeability of G˗ve bacteria can be analyzed quantitatively by fluorescent dyes 1‐*N*‐phenylnaphthylamine (NPN) and SYTOX Green, respectively. From Figure 4C, a concentration of 2 µm **D‐zp37** was sufficient to markedly destroy the integrity of the three studied *Vibrio* strains. Though the level of OM disruption was inferior to the positive control 0.1% Triton X‐100, **D‐zp37** still showed strong membrane‐active capability. SYTOX Green spectrums further validated that the IM of *Vibrio* bacteria were significantly impaired after the **D‐zp37** treatment (Figure 4D). Noteworthily, at least for *VA* 1579 and *VP* 1647, subjected to the treatment of 2 µm **D‐zp37** could lead to more serious IM damage than 0.1% Triton X‐100.

In addition to the physical structural damage of *Vibrio* bacteria, **D‐zp37** was demonstrated to impede the ETC. Using a voltage sensitive probe *3*, *3’*‐dipropylthiadicarbocyanine iodide [DiSC_3_(5)], we noticed that the fluorescence intensity ​​increased rapidly within seconds in the presence of 1 µm **D‐zp37** for all three *Vibrio* strains (Figure 4E). Compared with the acknowledged membrane potential modulator valinomycin (VAL), **D‐zp37** could induce approximately the same degree of potential dissipation at 2 µm for *VA* 1579, *VP* 1647 and 8 µm for *VV* 5043. Furthermore, in comparation with untreated bacterial cells, the intracellular adenosine triphosphate (ATP) level of tested *Vibrio* strains decreased significantly after 2 µm **D‐zp37** treatment (Figure 4F). Numerically, ATP contents of *VA* 1579, *VP* 1647 and *VV* 5043 all fell below 10% when the **D‐zp37** concentration increased to 8 µm and beyond.

Increased ROS levels were clearly detected when bacterial cells were exposed to **D‐zp37** (Figure 4G). At their respective 0.25‐fold MICs, ROS levels were 1.42 times higher for *VA* 1579, 1.24 times higher for *VP* 1647 and 1.21 times higher for *VV* 5043, indicating that sub‐MIC treatment could already result in an obvious oxidative stress response. When *Vibrio* cells were treated with 4‐fold MIC of **D‐zp37**, the fold change of ROS increased sharply to 3.52 (*VA* 1579), 2.36 (*VP* 1647) and 3.35 (*VV* 5043), respectively.

DNA of *Vibrio* strains were extracted and their interaction with **D‐zp37** was examined. As illustrated in Figure 4H, incubating with 5 µm **D‐zp37** led to slight tailing in agarose gel electrophoresis imaging. More significant DNA retardation was observed if higher concentrations of **D‐zp37** were used, hinting that **D‐zp37** was able to bind with DNA and cause aggregation. These results collectively depicted the multi‐mode antibacterial mechanisms of **D‐zp37** against *Vibrio* (Figure 4I).

### 2.5 Omics Studies Reveal that D‐zp37 Invalidates Membrane Stress Response and Blocks Amino Acid Biosynthesis of *Vibrio* Cells

**FIGURE 5:**
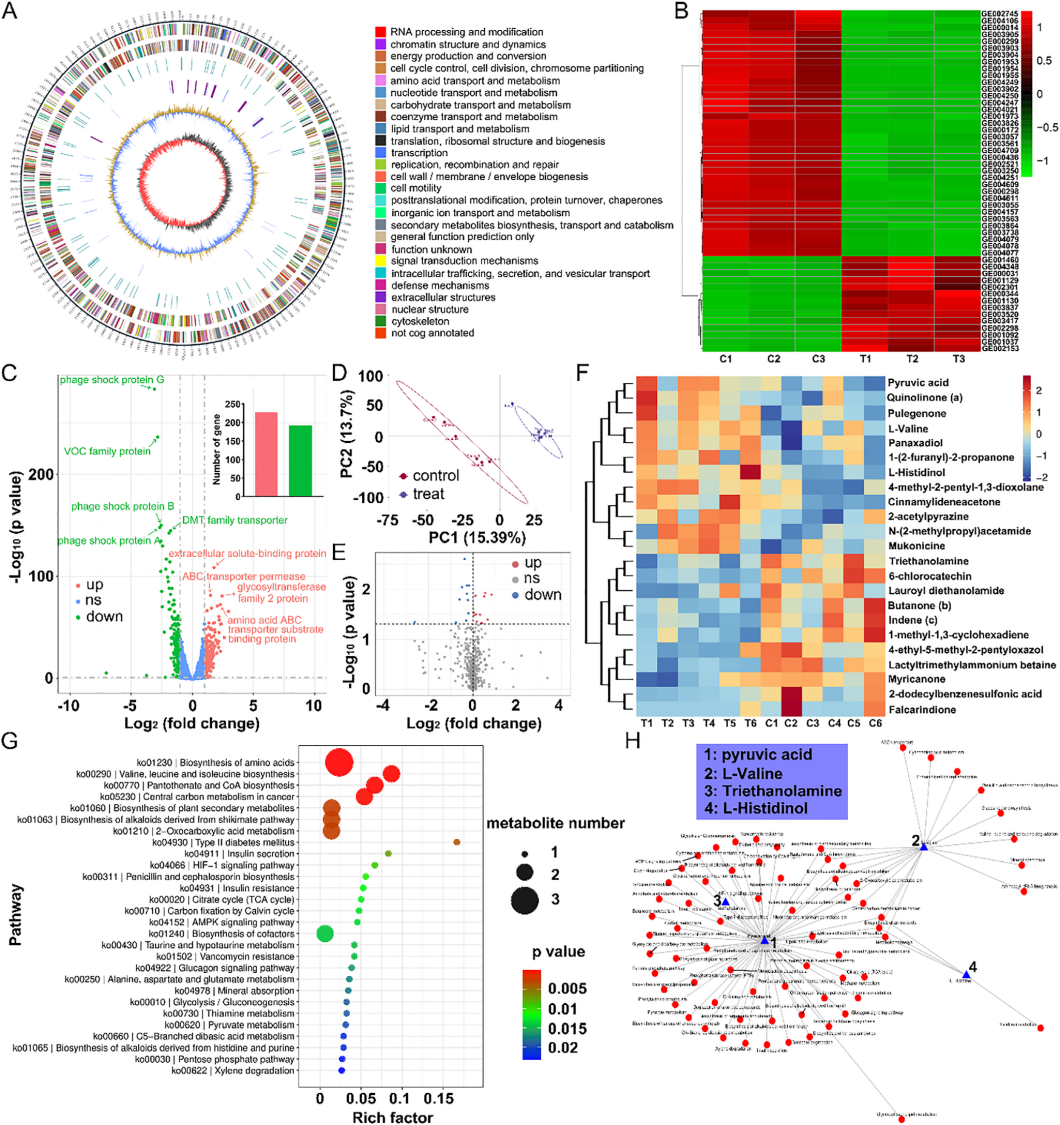
(A) Genomic circle map of *VA* 1579. Outermost circle: genome size marker. Second and third circles: genes on the plus strand and minus strand of the genome respectively. Different colours represent different clusters of orthologous genes functional classes. Fourth circle: repeat sequence. Fifth circle: tRNA is shown in blue and rRNA is shown in purple. Sixth circle: GC content. The region which GC content higher than the average GC content of the genome is shown in light yellow, the reverse is in blue. Innermost circle: GC‐skew, with dark gray representing regions where G content is greater than C and red representing regions where C content is greater than G. (B) Heat map of top 50 differential mRNA clustering. (C) Volcano plot of differential mRNA expression. Corresponding representative expressed proteins are annotated. (D) Partial least squares discriminant analysis between control and treat groups of non‐targeted metabolomics. (E) Volcano plot of differential metabolites. (F) Heat map of top 30 differential metabolites. Quinolinone (a): 2‐[4‐(3,4‐methylenedioxyphenyl) butyl]‐4(1H)‐quinolinone. Butanone (b): 4‐(2,6,6‐trimethyl‐1,3‐cyclohexadien‐1‐yl)‐2‐butanone. Indene (c): 5,7alpha‐dihydro‐1,4,4,7a‐tetramethyl‐4H‐indene. (G) KEGG enrichment scatterplot of non‐targeted metabolomics. Pathways with the top 20 smallest *p*‐values are displayed. (H) Network diagram of regulatory relationships between metabolites and pathways. Four key metabolites identified were shown in purple.

To further clarify the potential effects of **D‐zp37** on signalling pathways of *Vibrio* cells, we employed transcriptomics and metabolomics approaches on *VA* 1579 before and after **D‐zp37** treatment. *Vibrio* species, *VA* 1579, an environmentally isolated drug‐resistant strain showed the highest sensitivity to **D‐zp37** was chosen for detailed analysis. Figure 5A shows the genomic circle map, serving as a reference for downstream molecular investigations.

Gene ontology and EggNOG database annotation of *VA* 1579 are presented in Figures S2 and S3, respectively. Using the complete genome of *VA* 1579 as a reference, a total of 4990 genes were identified, and Figure 5B lists the top 50 differential mRNA clustering between control and **D‐zp37** treatment groups. Among them, 227 genes were upregulated, and 192 were downregulated (Figure 5C). Protein annotation of these genes highlights the potential involvement of the phage shock protein (PSP) family, a cell membrane stress protective system, in governing the antibacterial mechanisms of **D‐zp37**. Three members (pspG, pspB and pspA) were ranked as the top 5 downregulated genes, suggesting impaired membrane stress adaptation. In contrast, genes encoding ABC transporter permease were significantly upregulated in response to **D‐zp37** exposure, indicating enhanced membrane transport activity. In validating the transcriptomics analysis, qPCR was employed to quantify the expression levels of the interested genes (Figure S4). The expression of pspG, pspB, pspA and DMT genes were all significantly downregulated, while the ABC transporter permease was significantly upregulated, agreeing with sequencing findings. However, the expression levels of SBPs were found to be downregulated, disagreeing with transcriptomics results.

Next, metabolite changes were investigated. Classification annotation statistical charts of identified metabolites in the Human Metabolome Database and Kyoto Encyclopedia of Genes and Genomes (KEGG) pathways were presented in Figures S5 and S6, respectively. Partial least squares discriminant analysis (PLS‐DA) suggested that 6 biological repeats in each group had a similar expression pattern (Figure 5D). The number of differential metabolic ions (Figure S7) and differential secondary metabolites (Figure S8) before and after **D‐zp37** treatment was summarized. Volcano plot illustrated in Figure 5E demonstrated that among the secondary metabolites, 12 of them were upregulated and 11 were downregulated. The top 30 differential metabolites were displayed in a heat map (Figure 5F). Furthermore, KEGG enrichment revealed the possible biological functions of differential metabolites. According to Figure 5G, the amino acids biosynthesis of *VA* 1579, particularly valine, leucine and isoleucine, was significantly affected in the presence of **D‐zp37**. Based on KEGG pathway analysis of differential metabolites, a regulatory network diagram illustrating the relationships between key metabolites and associated pathways was constructed (Figure 5H). Four important metabolites, including pyruvic acid, L‐Valine, triethanolamine and L‐Histidinol, were highlighted due to their relevance in metabolic shifts. This network diagram effectively mapped the interactions between metabolic changes and pathway perturbation, highlighting the concerned pathways. Additionally, a correlation heat map revealing positive (shown in red) and negative (shown in blue) correlations between metabolites is presented in Figure S9, further supporting the systemic metabolic response induced by **D‐zp37**.

### 2.6 D‐zp37 Reduces Bacterial Load of *Vibrio* Mixture In Vivo

**FIGURE 6:**
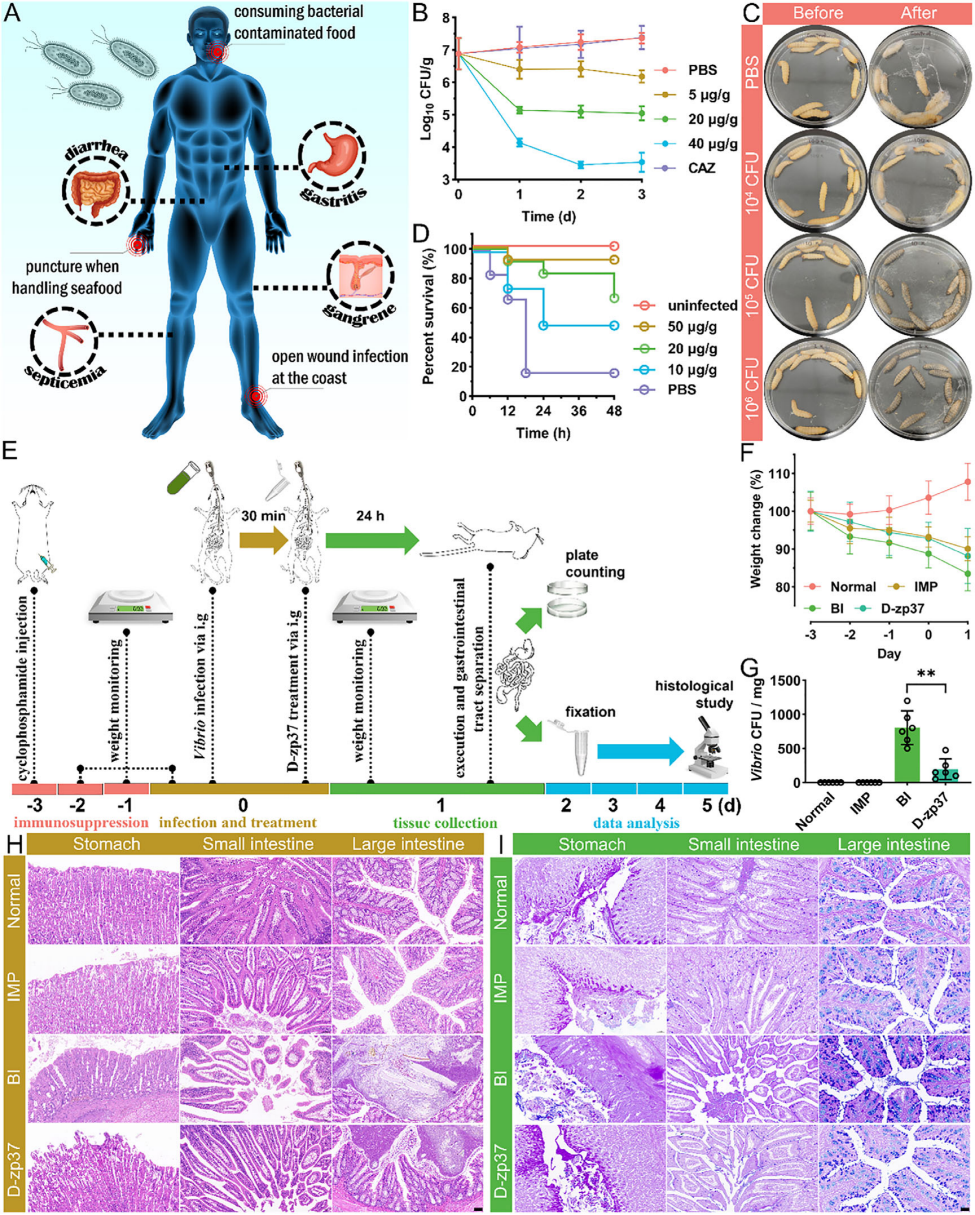
(A) Route of *Vibrio* infection and common vibriosis. (B) Live bacterial counts of infected shrimp samples during a 3‐day cold storage. (C) Virulence test of *Vibrio* mixture. (D) Survival ratio of infected larvae before and after **D‐zp37** treatment. (E) Flow diagram of mouse model for in vivo antibacterial efficacy and histological studies (*n* = 6 per treatment group). (F) Body weight change of mouse. (G) Total *Vibrio* counts in the intestinal tract of the mouse. (H) H&E staining images of four groups of mice. Scale bar: 50 µm. (I) AB‐PAS staining images of four groups of mice. Scale bar: 50 µm.

*Vibrio* can invade the human body via many routes, including from uncooked aquatic products, punctured wounds during seafood processing and seriously contaminated seawater [25], inducing gastritis, diarrhea and gangrene (Figure 6A). Here, we employed three infection models (shrimp, *Galleria mellonella* larvae and mouse) to comprehensively evaluate the antibacterial efficacy of **D‐zp37**.

Shrimps were infected with *Vibrio* mixture to simulate food contamination and then treated with **D‐zp37** and CAZ, respectively (Figure 6B). Time‐killing curves showed that when **D‐zp37** was applied, total *Vibrio* colonies declined during a cold storage test lasting 3 days. The plummet occurred on the first day, which was consistent with previous bacteriolytic data. As controls, the CFU of PBS‐treated and CAZ‐treated *Vibrio* tended to increase.

Next, *Galleria mellonella* larvae were used to preliminarily test the in vivo effect of **D‐zp37**. Three dosages (10^4^–10^6^ CFU per larva) of *Vibrio* mixture were injected to evaluate its virulence first (Figure 6C). After 12 h of infection, all individuals in the group infected with 10^5^ CFU exhibited obvious melanization, suggesting that this dosage could lead to considerable virulence to larvae. Subsequently, the survival status of *Galleria mellonella* was monitored to see whether **D‐zp37** could rescue infected larvae (Figure 6D). Inspiringly, the mortalities of **D‐zp37**‐treated groups were lower than those of the PBS‐treated group at each interval. After 48 h, the survival ratio of 50 µg/g group was >90%, far outperforming the PBS‐treated group (<20%).

*Vibrio* infections bring a high risk to people, especially the immunocompromised ones. We therefore established an immunosuppressed mouse model to further verify the antibacterial effect of **D‐zp37** in vivo (Figure 6E). Four mice groups were set and defined as: normal (healthy with normal feeding), immunosuppression (IMP, subjected to cyclophosphamide), bacterial infection (BI, subjected to cyclophosphamide, then *Vibrio* infection) and **D‐zp37** (subjected to cyclophosphamide, then *Vibrio* infection, followed with 50 µg/g **D‐zp37** treatment). Via intragastric (i.g) administration, mice were infected with high concentrations (OD_600_ = 0.3) of *Vibrio* mixture. Then, **D‐zp37** was given by the same approach. As shown in Figure 6F, the average body weight of mice in the normal group increased, while mice in the other three groups suffered from immunosuppression lose weight obviously. Even though mice in the BI and **D‐zp37** groups numerically had a greater weight loss compared with those in the IMP group, there was no statistical difference between these three groups. On the one hand, this indicated the successful establishment of an immunosuppressed model, and on the other hand, it suggested that the short‐term *Vibrio* infection (1 day) did not have much effect on the weight of mice.

Differently, the plate counting result revealed that in the intestine of mice, live colonies of *Vibrio* cells varied significantly (Figure 6G). After the *Vibrio* infection, the bacterial number increased from an undetected level to >800 CFU/mg. The **D‐zp37** treatment could reduce it to <200 CFU/mg. The capability of **D‐zp37** in killing *Vibrio* in the complex gastrointestinal environment was thus confirmed. Besides the bacterial load, histology of the stomach, small intestine and large intestine were performed to investigate possible pathological changes after *Vibrio* infection. Hematoxylin and eosin (H&E) staining revealed that bacterial infection induced pathological changes of tissues in the stomach and intestine (Figure 6H). Alcian blue and Periodic acid‐Schiff (AB‐PAS) staining suggested that the bacterial infection may lead to excessive secretion of acidic mucous substance. After **D‐zp37** treatment, some pathological features like organizational distortion were relieved (Figure 6I).

### 2.7 D‐zp37 Ameliorates *Vibrio* Invasion‐Associated Gut Microbiota Disequilibrium

**FIGURE 7:**
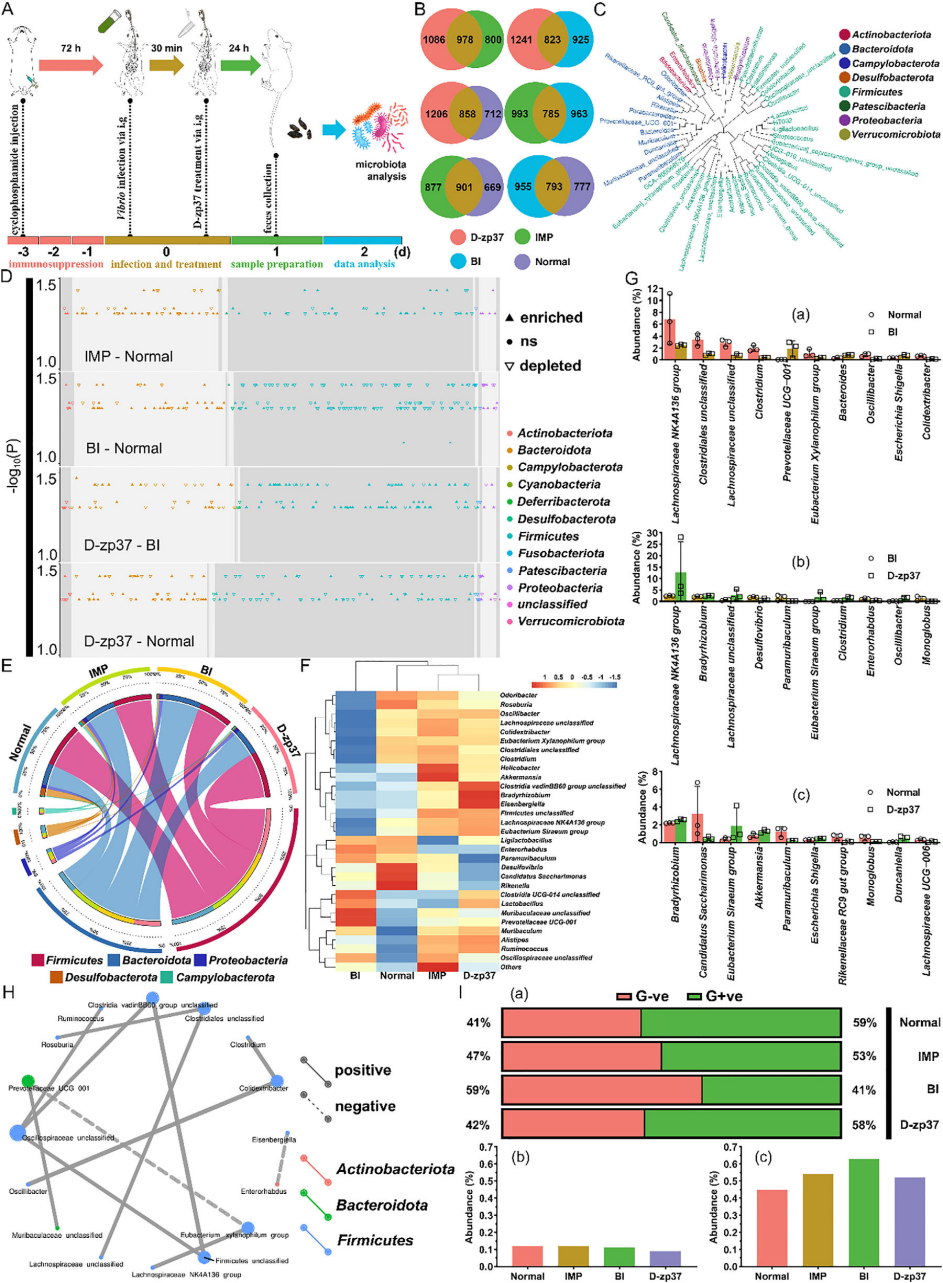
(A) Flow diagram of mouse model for gut microbiota analysis. (B) Venn plots of the number of ASVs. (C) Evolutionary tree of the top 50 abundance species. (D) Abundance changes of different species. (E) Microbiota variation between four groups in phylum level. (F) Microbiota variation between four groups in genus level. (G) Top 10 genera with a significant difference. (H) The correlation between pairwise dominant genera. (I) Phenotypes prediction including Gram staining category (a), biofilm forming (b) and potential pathogenicity (c), between four testing groups.

Bacterial invasion may trigger gut microbiota disequilibrium [26]. We then analysed the fecal microbiomics of mice after *Vibrio* infection and **D‐zp37** treatment (Figure 7A). Taking advantage of 16s rDNA‐based high‐throughput sequencing, microbial community composition in mouse fecal samples were studied. The Venn plots visually presented the number of amplicon sequence variants (ASVs) which were common or specific between each group (Figure 7B). The evolutionary tree illustrated in Figure 7C suggested that *Firmicutes* would be the dominant phylum, occupying 60% quota of the top 50 abundance species. Manhattan plots provided an overview of abundance change of different species between pairings (Figure 7D). For more details, the microflora of four groups in phylum level indicated that *Vibrio* infection could lead to ratio decrease of *Firmicutes* and increase of *Bacteroidota*, but **D‐zp37** treatment reversed the trend (Figure 7E). In genus level, the abundance of several fundamental anaerobic acid‐producing bacteria like *Odoribacter*, *Roseburia*, and *Oscillibacter* declined after infection (Figure 7F).

By analysing the top 10 genera with significant difference, it should be noticed that *Lachnospiraceae* NK4A136 depleted dramatically in the BI group (Figure 7G(a)) but enriched again in **D‐zp37** treatment group (Figure 7G(b)). There was no significant difference between the normal group and the **D‐zp37** group (Figure 7G(c)). One of the key roles of *Lachnospiraceae* would be the efficient fermenters of complex carbohydrates and dietary fibers. They produce short chain fatty acids (SCFAs), notably acetic acid, propionic acid and butyric acid. Subsequent experiments validated that *VA* infection would cause a significant decrease in the content of these SCFAs in the feces of mice. However, after **D‐zp37** treatment, their concentrations could be restored (Figure S10).

Spearman network diagram reported the correlation between pairwise dominant genera (Figure 7H). Here we identified strong positive correlations between *Oscillibacter* and *Colidextribacter*, *Clostridia* and *Firmicutes* species. Negative correlations were also detected between *Prevotellaceae* and *Eubacterium xylanophilum*, *Eisenbergiella* and *Enterorhabdus*. Finally, the phenotypes of four groups were predicted by BugBase [27]. The three most concerned phenotypes, namely Gram staining category, biofilm forming and potential pathogenicity, were shown here. Ratio of G‒ve bacteria increased from 41% (normal) to 47% (IMP) and 59% (BI), respectively, but **D‐zp37** treatment resulted in an obvious decrease to 42% (Figure 7I(a)). For the abundance of biofilm forming bacteria, these four groups didn't exhibit a significant difference (Figure 7(b)). However, the **D‐zp37** treatment was predicted to lower the relative abundance of potential pathogenic bacteria (Figure 7I(c)).

## 3 Discussion

Although presenting desired helical structure and amphiphilic interface, the poor proteolytic stability of **zp37** would certainly impose in vivo stability issues, limiting its potential clinical application. Non‐proteinogenic amino acids have unique advantages in resisting enzymatic degradation [28]. Thus, introducing unnatural residues is an efficient and widely used approach to prolong the in vivo half‐life of peptide drugs, maximizing their therapeutic effects [29]. Retaining the bioactivity of modified peptides is essential to ensure their therapeutic potential is not compromised. Based on the MIC data, **D‐zp37** exhibited comprehensively enhanced bioactivity against *Vibrio* compared to its precursor, **zp37**. The susceptibility of tested *Vibrio* strains, which were resistant to penicillins and cephalosporins [30], to **D‐zp37** suggested its potential as a promising antibacterial agent for overcoming antimicrobial resistance.

HPLC results suggested that chiral conversion confers **zp37** with improved stability. **D‐zp37** also retained its bioactivity under various environmental conditions, with only a slight reduction in the presence of 50% FBS, 10% GJ and 25% IJ, demonstrating its strong environmental tolerance. Furthermore, **D‐zp37** exhibited rapid bactericidal effects rather than bacteriostatic action, suggesting its potential for swift microbial eradication. Collectively, the improved proteolytic stability, broad‐spectrum bioactivity, and environmental resilience of the chirally‐converted peptide **D‐zp37** highlighted its potential as a potent antibacterial candidate against *Vibrio* species.

**D‐zp37** exerts its bactericidal effect by disrupting the bacterial membrane structure. Encouragingly, this adverse impact is mild in mammalian cells. The selectivity is likely due to the composition differences in the membrane surface. While *Vibrio* membranes are rich in negatively charged phospholipids, mammalian cells predominantly contain neutral phospholipids [31]. After destroying the integrity of *Vibrio*’s membrane, **D‐zp37** can be internalized into the bacterial cytoplasm, further enhancing its antibacterial efficiency via an intracellular bactericidal process. The ability of **D‐zp37** to selectively lyse *Vibrio* cells without affecting the coexisting human digestive tract cells lays a strong foundation for its potential application in treating *Vibrio*‐induced gastrointestinal polymicrobial infections.

The rapid motility of *Vibrio* cells, facilitated by flagella, contributes to the rapid dissemination of drug‐resistant genes and enhanced difficulty of prevention and control of the associated infections. The reduction of bacterial swimming area indicates that the presence of **D‐zp37** can partially inhibit the spread of bacteria. This may be partly due to the direct bactericidal activity of **D‐zp37**, resulting in a decrease in the total number of bacteria participating in swimming behaviour. In addition, drug treatment may influence the physiological activities of bacteria, such as downregulating the expression of genes related to motility, impairing the flagellar function and movement [32].

Bacteria embedded within biofilms exhibit significantly greater resistance to antibacterial agents compared to their planktonic counterparts. In this study, we proposed that **D‐zp37** may inhibit the efflux of polysaccharide from the interior to the exterior of *Vibrio* cells, leading to aberrant intracellular accumulation of polysaccharides. This disruption likely interferes with the extracellular matrix assembly, thereby delaying biofilm formation. Furthermore, **D‐zp37** demonstrated the ability to disperse pre‐established biofilms, indicating its dual functionality in both prevention and eradication of biofilm‐associated *Vibrio* populations.

The majority of cationic AMPs exert their antibacterial activity via targeting the bacterial membranes [33]. Their positively‐charged functional groups, such as amino or guanidine moieties, interact with negatively‐charged membrane components of G˗ve bacteria, particularly LPS, to disrupt essential physiological functions of bacteria [34]. In the case of **D‐zp37**, reduced antibacterial activity in the presence of exogenous LPS (Figure 4B) can be reasonably attributed to the sequestration of peptide molecules by free LPS, lowering the effective concentration of **D‐zp37** available to act on the bacterial membrane and diminishing its killing efficiency. Membrane damage caused by AMPs is typically characterized by an increase in permeability [35]. Our results indicated that **D‐zp37** compromises the proton motive force associated with ETC, thereby impeding energy production. This is supported by a marked decline in intracellular ATP levels (Figure 4F), indicating severe ETC dysfunction. Additionally, **D‐zp37** induced excessive intracellular ROS accumulation, posing a critical threat to bacterial viability by oxidizing and irreversibly damaging essential cellular components [36].

Transcriptomics analysis further revealed that **D‐zp37** disrupts the membrane stress response in *Vibrio* by impairing the PSP system, which is vital for maintaining cell viability under stress [37]. The downregulation of PSP‐related genes indicated compromised inner membrane integrity and a failure to initiate effective repair mechanisms. Concurrently, the significant upregulation of the gene encoding ABC transporter permease hinted enhanced membrane transport activity, possibly as a compensatory response to stress. This overexpression may also increase ATP consumption and imposed additional strain on membrane function. It is worth noting that qPCR data suggested the expression levels of SBPs were downregulated, contradicting the transcriptomics results. This discrepancy may reflect differences in temporal expression dynamics, post‑transcriptional regulation, or methodological sensitivity. Alternatively, D‑zp37 may trigger a rapid but transient activation of transporter permease genes while ultimately suppressing solute‑binding components as membrane damage progresses. Together, these findings suggest that **D‑zp37** induces a multifaceted collapse of membrane homeostasis, disrupting both protective stress pathways and nutrient transport systems, contributing to reduced cellular resilience and promoting bacterial susceptibility.

Complementary metabolomics data showed that **D‐zp37** treatment led to significant metabolic shifts in *Vibrio*, particularly those involved in amino acid biosynthesis. The metabolic disruptions are likely to reflect as downstream consequences of the transcriptomic changes. Impaired membrane function and energy depletion (evidenced by reduced ATP levels) hindered biosynthetic processes, while stress‐induced transcriptional reprogramming may suppress genes involved in metabolic homeostasis, further contributing to the altered metabolite profile.

The integrated analysis of molecular biological assays, transcriptomic profiles and metabolomic shifts present a consistent and multifaceted mechanism underlying the antibacterial activity of **D‐zp37**. Each dataset independently highlights disruptions in membrane integrity, energy metabolism, and stress response pathways. Together, they form a coherent picture of how the peptide compromises *Vibrio* viability. Structural damage observed through the biological assay is reflected in transcriptomic evidence of impaired membrane repair and metabolic shifts in biosynthetic functions, particularly amino acid metabolism. This alignment reinforces the reliability of the proposed mechanism: **D‐zp37** initiates its lytic action by interacting with LPS on the outer membrane of *Vibrio* cells, compromising the membrane integrity and enabling entry into the cytoplasm. Once inside, the peptide disrupts the proton motive force and the ETC, leading to dissipation of membrane potential and reduced ATP synthesis. It also interacts with DNA, triggering abnormal intracellular accumulation of ROS, which oxidize and damage essential cellular components. The cumulative effects contribute to the rapid and effective killing of *Vibrio* cells. Importantly, this multifaceted mode of action may reduce the likelihood of resistance development as it targets multiple essential pathways simultaneously, making **D‐zp37** an attractive antibacterial candidate.

Building on the mechanistic insights, we next evaluated the therapeutic potential of **D‐zp37** in living systems. The effectiveness of **D‐zp37** in controlling foodborne contamination was first demonstrated using a shrimp model, confirming its applicability in real‐world food environments. Then, the in vivo curative effect of **D‐zp37** was confirmed in a larvae infection model, where **D‐zp37** treatment significantly improved the survival rates of larvae infected with a lethal dose of *Vibrio* bacteria. Encouraged by these results, a mouse model was employed to further assess the in vivo efficacy of **D‐zp37**. After intragastric administration, the number of live *Vibrio* bacteria in the intestine was significantly reduced, indicating that **D‐zp37** retained its antibacterial activity even within the complex digestive tract environment.

Fecal microbiome analysis revealed that *Vibrio* infection disrupted the synthesis of short‐chain fatty acids, primarily produced by anaerobic acid‐producing bacteria like *Odoribacter*. These metabolites play a vital role in maintaining intestinal health, and their reduction reflects a disturbance in gut microbial balance. For mice treated with **D‐zp37**, the abundance of *Odoribacter* and other SCFA‐producing strains was restored to baseline levels, suggesting that **D‐zp37** contributes to the recovery of beneficial microbiota. Further supporting this observation, the abundance of *Lachnospiraceae*, which is a carbohydrate‐metabolizing bacterial family known for its role in promoting gut homeostasis [38], also returned to normal levels after **D‐zp37** treatment. This indicates that **D‐zp37** not only combats *Vibrio* infection but may also help preserve or restore the functional integrity of the gut ecosystem. Interestingly, no detectable *Vibrio* cells were found in the microbiome analysis, likely due to their low abundance relative to dominant endogenous gut bacteria. Collectively, these findings suggest that while *Vibrio* infection can compromise digestive health by disturbing microbial composition and metabolic output, **D‐zp37** effectively mitigates these effects and supports the restoration of a healthy intestinal microbiota.

## 4 Materials and Methods

### 4.1 Peptide Characteristics and Antibacterial Activity

#### 4.1.1 Cleavage Sites Prediction

Two peptides were customized from Synpeptide (Nanjing, China). The quality control result of **D‐zp37** was shown in **Figure** **S11**. Potential cleavage sites of **zp37** were predicted by the website Expasy (https://www.expasy.org/resources/peptidecutter).

#### 4.1.2 Structure Prediction

Secondary structure and Van der Waals surface diagram were generated by PEP‐FOLD 4 (https://bioserv.rpbs.univ‐paris‐diderot.fr/services/PEP‐FOLD4/).

#### 4.1.3 MIC Determination

All *Vibrio* strains used in this study were isolated from the environment and stored in our lab. MIC values of **zp37** and **D‐zp37** against them were determined by a classic double‐dilution assay. In brief, bacterial suspensions (5 × 10^5^ CFU/mL) in Mueller Hinton Broth (MHB, Hopebio, China) were treated with peptides at different concentrations for 18 h in a 96‐well plate at 37°C. The lowest concentration without observing bacterial turbidity was defined as its MIC.

#### 4.1.4 Proteolytic Stability in Trypsin

Peptide solutions of **zp37** and **D‐zp37** (100 µm) were incubated with 10 µg/mL trypsin (HyClone, USA). Peak areas were monitored at 30 min intervals using HPLC (1200 infinity series, Agilent Technologies, USA). Peak area at 0 min was defined as 100%.

#### 4.1.5 Inhibition Effect in Different Conditions

Selected *Vibrio* cells were inoculated in a 96‐well plate with an initial density of 5 × 10^5^ CFU/mL in the absence or presence of specified additives. Trypsin and FBS were purchased from HyClone, USA. Simulated GJ and IJ were purchased from Chuang Feng Technology, China. NaCl was purchase from Sigma‐Aldrich, USA. After 6 h incubation at 37°C, OD_600_ values were measured (Clariostar, BMG Labtech, Germany). The turbidity value of bacteria incubated in the MHB medium was defined as 100% growth.

#### 4.1.6 Time Killing Test

Selected *Vibrio* cells (approximately 10^6^ CFU/mL) were treated with PBS (Solarbio, China) or **D‐zp37** at their respective MIC, 2‐fold MIC and 4‐fold MIC. A volume of 10 µL bacterial suspension was sampled to perform plating counting in Mueller Hinton agar (Hopebio, China) at 20 min intervals, lasting 60 min.

### 4.2 Bioactivity to *Vibrio* Mixture and Investigation on Cell Selectivity

#### 4.2.1 Turbidity Test

OD_600_ values of three *Vibrio* strains (*VA* 1579, *VP* 1647 and *VV* 5043) were adjusted to approximately 1.0 individually in PBS. Then, they were mixed with the ratio of 1:1:1. Homogeneous polymicrobial suspension was divided into six groups and treated with PBS, 1–8 µm **D‐zp37** or 8 µM CAZ (Sigma–Aldrich, USA) respectively. Turbidity changes of each group were monitored by measuring the OD_600_ values every 15 min by a biophotometer (D30, Eppendorf, Germany).

#### 4.2.2 SEM

Homogeneous polymicrobial suspensions prepared as above were treated with PBS, 2 or 8 µM **D‐zp37** respectively for 1 h at 37°C. Afterward, bacterial cells were fixed with 2.5% glutaraldehyde (Tokyo Chemical Industry, Japan) overnight at 4°C. Then, samples were immersed into a graded ethanol (Anaqua Chemicals Supply, USA) series from 50%, 70%, 90%, to 100% in sequence (10 min each grade). After resuspending them in 100% ethanol again, a volume of 2 µL suspension was dropped on a sterile cover glass. Finally, samples were air‐dried, coated with platinum, and imaged using a SEM facility (FEI Quanta 400F, Philips, Netherlands).

#### 4.2.3 Hemolysis

Fresh blood was obtained from a healthy mouse. After centrifugation with 4000 rpm (Universal 320R benchtop centrifuge, Tegent, Hong Kong) for 10 min, the supernatant was discarded and the remaining red blood cells (RBCs) were diluted to 8% with PBS. RBCs were treated with PBS, **D‐zp37** or 1% Triton X‐100 for 1 h, respectively. Cells were then centrifuged again at 4000 rpm for another 10 min. The absorbance of supernatant at 540 nm was measured (Clariostar, BMG Labtech, Germany). RBCs treated with PBS and 1% Triton X‐100 were set as negative control (0% hemolysis) and positive control (100% hemolysis) respectively.

#### 4.2.4 Triple Fluorescence Staining

**FITC‐D‐zp37** was customized from Synpeptide (Nanjing, China) with >95% purity (Figure S12). Homogeneous polymicrobial suspensions prepared as above was diluted to an OD_600_ value at 0.5. Bacterial cells were stained by 10 µg/mL DAPI (Thermo Fisher, USA) for 20 min, followed by 10 µM **FITC‐D‐zp37** treatment. At specified timepoints (15, 30 and 60 min), part of cells was sampled, centrifuged to remove unbound dyes and resuspended in PBS containing 25 µm PI (Invitrogen, USA) for another 20 min staining. Subsequently, cells were rinsed thrice and fixed with 2.5% glutaraldehyde for 1 h. Prepared samples were dropped on a sterile coverslip and imaged by a live‐cell fluorescence imaging system (Eclipse Ti2E, Nikon, Japan).

#### 4.2.5 TEM

Homogeneous polymicrobial suspensions prepared as above were treated with PBS and **D‐zp37** at various concentrations for 1 h. Then, cells were subjected to dual fixation. The compositions of the prefixation solution included 2% formaldehyde (Sigma–Aldrich, USA), 2.5% glutaraldehyde, 0.075% ruthenium red (Sigma–Aldrich, USA) and 0.075 m lysine‐acetate (Sigma–Aldrich, USA) in cacodylate buffer (Sigma–Aldrich, USA) with pH at 6.9. After 20 min prefixation, bacterial cells were rinsed thrice with cacodylate buffer containing 0.075% ruthenium red and fixed again with the lysine‐acetate‐free mixture overnight at 4°C. On the next day, secondary fixation was performed using the solution composed of 1% OsO_4_ (Sigma–Aldrich, USA) and 0.075% ruthenium red in cacodylate buffer for another 1 h. Finally, the samples were dehydrated with a series of graded ethanol solutions. After resin embedding, and ultrathin sectioning, TEM (HT7700, Hitachi, Japan) pictures were taken with 12000‐ and 30000‐fold magnification.

#### 4.2.6 Cytotoxicity

GES‐1 cells seeded in a 96‐well plate were incubated with **D‐zp37** at various concentrations for 24 h, followed by adding 10 µL of CCK‐8 solution (Solarbio, China) for another 4 h incubation. Next, the absorbance values at a wavelength of 450 nm were measured using a microplate reader (Clariostar, BMG Labtech, Germany). GES‐1 cells treated with PBS was defined as 100% survival.

#### 4.2.7 Co‐Culture Image

A sterile coverslip was placed at the bottom of a 6‐well plate. GES‐1 cells in Dulbecco's modified eagle medium (DMEM) containing 10% FBS were seeded. After 24 h incubation, Nile red (Solarbio, China) at 10 µg/mL was then employed to stain GES‐1 cells for 30 min. Subsequently, DMEM was replaced by a homogeneous polymicrobial suspension with an OD_600_ value of 0.3 for 10 min infection. Next, the bacterial solution was gently removed and such a co‐culture system of *Vibrio* and GES‐1 was treated with 10 µg/mL Hoechst 33258 (Solarbio, China) for 30 min and 20 µM **FITC‐D‐zp37** for another 30 min in succession. Afterward, *Vibrio* cells were collected and rinsed thrice. In the meanwhile, GES‐1 cells were also rinsed thrice. The coverslip, whose upper surface was covered with GES‐1 cells, was taken out and a total of 2 µL washed *Vibrio* solution was dropped on top of that. Finally, the photos were filmed by a live‐cell fluorescence imaging system (Eclipse Ti2E, Nikon, Japan).

### 4.3 Motility and Biofilm Related Studies

#### 4.3.1 Swimming

The 0.5% Luria–Bertani (LB) agar (Hopebio, China) plates containing different concentrations of **D‐zp37** (0–32 µm) were prepared in advance. A volume of 10 µL homogeneous polymicrobial suspension with OD_600_ at 0.1 was dropped on the center gently. Swimming pictures from day 1 to day 6 were captured by the ChemiDoc Imager (Bio–Rad, Hercules, USA). The longest straight‐line distance through the center was defined as the swimming diameter.

#### 4.3.2 Exopolysaccharide (EPS) Content Analysis

Homogeneous polymicrobial suspension (OD_600_ = 0.2) was divided into four groups and seeded in a black 96‐well plate. *Vibrio* mixtures were then treated with PBS and 2, 8 or 16 µm **D‐zp37** for 24 h, respectively. Planktonic cells were removed and wells were rinsed thrice. Next, the sediment was stained with 20 µg/mL concanavalin A conjugated by Alexa Fluor 488 (Thermo Fisher, USA) for 30 min to label EPS. Qualitative images were filmed by a live‐cell fluorescence imaging system (Eclipse Ti2E, Nikon, Japan) and the quantitative analysis was performed by a microplate reader (Clariostar, BMG Labtech, Germany).

#### 4.3.3 Intracellular Polysaccharide Content Analysis

Similar with the protocol described above, homogeneous polymicrobial suspension (OD_600_ = 0.2) was treated with PBS and 1, 2, 4 or 8 µm **D‐zp37** for 4 h, respectively. Cells were then centrifuged and bacterial pellets were collected. They were resuspended in equivalent PBS containing 20 µg/mL concanavalin A conjugated by Alexa Fluor 488 for 30 min staining. After that, OD_600_ values and fluorescence units were measured (Clariostar, BMG Labtech, Germany).

#### 4.3.4 Biofilm Inhibition

Homogeneous polymicrobial suspensions (initial total CFU was 5 × 10^5^ per mL) were treated with different concentrations of **D‐zp37** for 18 h in a 96‐well plate (150 µL per well). Planktonic cells were gently removed, and all wells were washed three times by deionized water. Then, biofilms were stained with 0.1% crystal violet (CV) solution (Sigma‐Aldrich, USA) for 30 min. After rinsing, a volume of 150 µL of absolute ethanol was added to dissolve the CV bound to biofilms. The control group without **D‐zp37** treatment was defined as 100%. The relative amount of biofilm for each group was calculated by comparing the absorbance at 595 nm with the control group. Five biological repeats were performed in this study.

#### 4.3.5 Biofilm Eradication

Homogeneous polymicrobial suspensions (initial total CFU was 5 × 10^5^ per mL) were seeded in a 96‐well plate and incubated at 37°C for 24 h. After removing planktonic bacterial cells, sediments were rinsed gently followed by 6 h **D‐zp37** treatment (0–64 µm). Phase images were subsequently filmed (Eclipse Ti2E, Nikon, Japan).

### 4.4 Antibacterial Mechanism

#### 4.4.1 Zeta Potential

*Vibrio* cells at logarithmic phase were collected and resuspended in PBS to OD_600_ at 0.2. Then, cells were subjected to **D‐zp37** (0‒8 µm) treatment for 10 min. The zeta potential of each sample was measured by Delsa Nano C particle analyser (Beckman Coulter, USA).

#### 4.4.2 Growth Kinetics in the Presence of LPS

LPS of *VA* 1579, *VP* 1647 and *VV* 5043, was extracted following the instructions of the specification of LPS extraction kit (Bestbio, China). Subsequently, bacterial suspensions of three *Vibrio* cells (OD_600_ = 0.1) were divided into four groups and treated with PBS, 10, 20 and 40 µg/mL corresponding LPS, respectively. The OD_600_ values were recorded continuously for 4 h (Clariostar, BMG Labtech, Germany).

#### 4.4.3 ITC

**D‐zp37** (200 µg/mL) and LPS (20 µg/mL) were dissolved in water, respectively. **D‐zp37** solution was then titrated into LPS solution following the handbook of Malvern MicroCal PEAQ‐ITC Automated Ultrasensitive Isothermal Titration Calorimeter (Malvern Instruments, UK). The operation parameters were set as –19 injections, 0.5 µL (first) and 2 µL (subsequent) for each injection volume, stir rate at 750 rpm, and temperature at 25°C.

#### 4.4.4 NPN Assay

*Vibrio* cells (OD_600_ = 0.6) were treated with **D‐zp37** at various concentrations (0.5‒32 µm) for 1 h. Cells treated with PBS and 0.1% Triton X‐100 (Solarbio, China) were set as two control groups. Then, bacterial suspensions were stained by 10 µm NPN (Sigma–Aldrich, USA) for 5 min and the fluorescence units were tested with an exciting wavelength at 350 nm and an emission wavelength at 420 nm (Clariostar, BMG Labtech, Germany).

#### 4.4.5 SYTOX Green assay

Membrane permeability was monitored following the instructions of the specification provided by the vendor (Mao Kang biotechnology, China) with slight modification. Briefly, *Vibrio* cells (OD_600_ = 0.6) were treated with PBS, **D‐zp37** or 0.1% Triton X‐100 respectively for 1 h. Next, SYTOX Green (5 µm) was applied to stain for 10 min at room temperature. Emission spectrums from 500 to 600 nm were recorded under the excitation wavelength at 488 nm (Clariostar, BMG LABTECH, Germany).

#### 4.4.6 Membrane Potential

*Vibrio* cells at the logarithmic phase were collected and adjusted to OD_600_ value of 0.3 in PBS. Then, cells were incubated with 0.5 µm potentiometric probe DiSC_3_(5) (Thermo Fisher, USA) for 1 h. Next, they were subjected to the treatment of PBS, **D‐zp37** (1–32 µm) and VAL (10 µg/mL), respectively. After that, the change of fluorescence units was instantly recorded with an excitation wavelength of 610 nm and an emission wavelength of 660 nm (Clariostar, BMG Labtech, Germany).

#### 4.4.7 ATP Level

*Vibrio* cells at the logarithmic phase were collected and adjusted to OD_600_ value of 0.5 in PBS. Cells were then treated with PBS or **D‐zp37** at various concentrations for 1 h. After that, cells were lysed and the relative ATP levels were determined by ATP assay kit (S0026, Beyotime, China) following the instructions of the specification. The luminescence value emitted by PBS‐treated group was defined as 100%.

#### 4.4.8 ROS Accumulation

*Vibrio* cells were resuspended in PBS with an adjusted OD_600_ value of 0.6. The cells were divided into six groups and treated with 0.25‐fold, 0.5‐fold, 1‐fold, 2‐fold, 4‐fold MIC of **D‐zp37**, and PBS as the control for 1 h respectively. Next, cells were stained with 10 µM *2, 7*‐dichlorodi‐hydrofluorescein diacetate (DCFH‐DA, Thermo Fisher, USA) for 30 min. After thrice rinse, fluorescence units were recorded with an exciting wavelength of 488 nm and an emission wavelength of 525 nm (Clariostar, BMG Labtech, Germany).

#### 4.4.9 DNA Binding

DNA of three *Vibrio* strains was obtained by DNA extraction and purification kits (Thermo Fisher, USA). They were then mixed with an equal volume of **D‐zp37** solutions and the final concentrations of **D‐zp37** were set as 0, 5, 10, 20, 40 and 80 µm respectively. After 1 h incubation, DNA shift was measured by 0.8% agarose gel electrophoresis.

### 4.5 Transcriptomics and Metabolomics

#### 4.5.1 Whole‐Genome Sequencing

*VA* 1579 was isolated and purified. Cells at logarithmic phase were sent to Shenzhen SMQ group medical laboratory (Shenzhen, China) for sequencing using PacBio technology.

#### 4.5.2 Transcriptomics and Real‐Time PCR Analysis

*VA* 1579 cells were grown to the logarithmic phase (OD_600_ = 0.6) and treated with PBS or **D‐zp37** (1 µm) for 2 h. Cells were then collected and sent to Shenzhen SMQ group medical laboratory (Shenzhen, China) for transcriptomics analysis. The expression level of genes identified to be significantly altered were quantified with Real‐Time qPCR. Total RNA was extracted from treated cells using the StarSpin Fast Cell RNA Kit (Genstar), following the manufacturer's instructions. Removal of genomic DNA contamination and cDNA synthesis were performed simultaneously with StarScript Pro All‐in‐one RT Mix with gDNA Remover Kit (Genstar, Beijing, China). The synthesized cDNAs was then quantified with RealStar Fast Pro SYBR qPCR Mix Kit (Genstar, Beijing, China) on Roche LightCycler 480 II Real‐Time PCR System equipment (F. Hoffmann‐La Roche Ltd, Basel, Switzerland) with primers detailed in Table S1. Data were normalized with the endogenous RpoD gene [39], which encode RNA polymerase sigma factor. The relative gene expression levels were calculated using the 2–ΔΔCt relative quantification method [40]. All experiments were repeated in triplicate.

#### 4.5.3 Metabolomics

*VA* 1579 cells at logarithmic phase (OD_600_ = 0.6) were divided into two groups (6 biological repeats per group). One was treated with 1 µm **D‐zp37** for 1 h, another was treated with equivalent PBS. Cells were then centrifuged to collect and washed with PBS thrice. After that, *Vibrio* cells were resuspended in −80°C methanol and stored at −20°C overnight. Finally, cells were centrifuged at 12000 rpm for 15 min. Supernatant was collected and sent to LC Biotechnology CO., Ltd (Hangzhou, China) for metabolomics analysis.

### 4.6 In Vivo Antibacterial Efficacy

#### 4.6.1 Shrimp Model

Live shrimps (*Trachypenaeus curvirostris*) were purchased from Tai Po market in Shatin. They were immersed in the homogeneous polymicrobial suspension (OD_600_ = 0.6) for 30 min for *Vibrio* infection. Shrimps were then divided into four groups and **D‐zp37** or CAZ (40 µg/g) were added. Samples were mixed evenly and stored at 4°C. At indicated intervals, an aliquot of shrimps (2 g) was transferred into 10 mL of sterile PBS and subjected to adequately grind. CFUs of total *Vibrio* cells in prepared homogenate was calculated by plate counting assay in the selective thiosulfate‐citrate‐bile salts‐sucrose (TCBS) agar (Hopebio, China).

#### 4.6.2 Vibrio Virulence

*Galleria mellonella* larvae (0.3‒0.4 g) were randomly divided into four groups. A volume of 10 µL PBS or homogeneous polymicrobial suspensions (10^4^, 10^5^ and 10^6^ CFU) were injected into larvae via the final prolegs. After 12 h incubation at 37°C, the survival ratio was recorded.

#### 4.6.3 Antibacterial Efficacy in Galleria Mellonella Model

Except for the control group, *Galleria mellonella* larvae were injected with 10^5^ CFU of homogeneous polymicrobial suspension. After 1 h infection, larvae were treated with PBS or **D‐zp37**. Survival ratios were determined at the specific time‐point. Each group had 12 larvae.

#### 4.6.4 Antibacterial Efficacy and Histological Analysis in Mouse Model

The mouse experiments were approved by the animal ethics committee, the Hong Kong Polytechnic University (approval number 23‐241645‐FSN‐R‐GRF). Female BALB/c mice (18–20 g) were divided into four experimental groups (*n* = 6): (1) Normal group, consisting of healthy mice; (2) IMP group, with mice subjected to immunosuppression without *Vibrio* infection; (3) BI group, with mice undergoing immunosuppression followed by *Vibrio* infection; (4) **D‐zp37** group with mice undergoing immunosuppression and *Vibrio* infection, followed by treatment with 50 µg/g **D‐zp37**. Immunosuppression was achieved by injecting 250 µg/g cyclophosphamide (Meryer, China) intraperitoneally (IMP, BI and **D‐zp37** groups). Three days later, a volume of 100 µL homogeneous polymicrobial suspension (OD_600_ = 0.3) was given to mice intragastrically (BI and **D‐zp37** groups). After 1 h infection, 50 µg/g **D‐zp37** was applied to treat **D‐zp37** group of infected mice intragastrically. For mice from other groups, they were given equivalent volume of PBS.

Body weight of all mice was constantly monitored from the day of performing immunosuppression to one day after **D‐zp37** treatment. Then, mice were sacrificed, and the large intestine was separated, subsequently homogenized. Total CFU of *Vibrio* were counted using TCBS agar. In the meanwhile, gastrointestinal tissues including stomach, small intestine and large intestine were fixed in 4% paraformaldehyde (Sigma–Aldrich, USA) and sent to Servicebio Technology (Wuhan, China) for histological analysis based on H&E and AB‐PAS staining.

### 4.7 Gut Microbiota

#### 4.7.1 Fecal Sample Collection

Mice were divided into four experimental groups (*n* = 3): (1) Normal group, consisting of healthy mice; (2) IMP group, with mice subjected to immunosuppression without *Vibrio* infection; (3) BI group, with mice undergoing immunosuppression followed by *Vibrio* infection; (4) **D‐zp37** group with mice undergoing immunosuppression and *Vibrio* infection, followed by treatment with 50 µg/g **D‐zp37**. For BI and **D‐zp37** groups, a homogeneous polymicrobial suspension was adjusted to OD_600_ value at 0.2. Then the bacterial mixture was diluted by 20‐fold in PBS. Mice were then infected with a volume of 100 µL of the diluted bacterial mixture intragastrically. For **D‐zp37** group, another 50 µg/g **D‐zp37** was given after 30 min infection. 24 h later, when mice defecated, fresh fecal samples were collected from the rectal area directly and cryopreserved at −80°C.

#### 4.7.2 Gut Microbiota

Gut microbiota was analysed by Biotree, Shanghai using 16S rDNA sequencing technology. DNA from four fecal samples was extracted, followed by PCR amplification. Purified samples were finally sequenced on the NovaSeq platform (Illumina, USA) according to the manufacturer's recommendations.

#### 4.7.3 SCFA Concentration

The fecal samples from four mice groups were collected. The acetic acid, propionic acid and butyric acid with known concentrations were used to draw the standard curves. Then, a volume of 2 mL of water (1:3 phosphoric acid solution) was added to each fecal samples, followed by vortex and homogenize for 2 min. Subsequently, 1 mL of ether was added for extraction for 10 min. Samples were centrifuged (4000 rpm, 20 min, 4°C). Another 1 mL of ether was supplemented for an additional 10 min centrifugation. Liquids from two extraction steps were collected, mixed and evaporated to less than 1 mL for analysis by gas chromatography‐mass spectrometer (GC‐MS, Thermo Scientific ISQ LT, USA).

### 4.8 Statistical Analysis

The results were expressed as mean ± standard error. Unless specified, three biological repeats were performed in all experiments. Original data were analysed by ANOVA and t‐test, where ns suggested no significant difference, ^*^ *p* < 0.05, ^**^ *p* < 0.01, ^***^ *p* < 0.001, and ^****^ *p* < 0.0001.

## 5 Conclusion

In this work, a new therapeutic peptide, namely **D‐zp37**, against polymicrobial infection caused by *Vibrio* superbugs was proposed. This novel peptide employed a simple chirality conversion strategy to improve its antibacterial activity and proteolytic stability. It could lyse *Vibrio* cells rapidly without affecting co‐existed normal cells. Mechanistic studies revealed that **D‐zp37** impeded ETC and could restrain the biofilm establishment of *Vibrio* cells via inhibiting the efflux of intracellular polysaccharides. Omics studies further suggested that **D‐zp37** may invalidate membrane stress response and blocks amino acid biosynthesis of *Vibrio* cells. In addition, the excellent antibacterial efficacy of **D‐zp37** was verified in three different infection models, including a shrimp contamination, a *Galleria mellonella* larvae infection and an immunosuppressed mice intestinal infection model. The intragastric administration of **D‐zp37** was further found to restore the imbalanced gut microbiota of immunosuppressed mice induced by polymicrobial *Vibrio* invasion. Collectively, these findings could be beneficial to people who are suffering from refractory *Vibrio* polymicrobial infections, especially those coastal residents with compromised immune systems.

## Author Contributions

**Ping Zeng**: Conceptualization, Methodology, Formal analysis, Investigation, Visualization, Writing – original draft. **Qipeng Cheng**: Methodology, Formal analysis, Investigation, Funding acquisition. **Xiaoxu Zhang**: Methodology, Investigation. **Honglan Wang**: Methodology, Investigation. **Pengfei Zhang**: Methodology, Investigation. **Jinhan Zhang**: Methodology, Investigation. **Xinyi Ding**: Validation, Investigation. **Lanhua Yi**: Methodology. **Kwok‐Yin Wong**: Resources. **Kin‐Fai Chan**: Resources. **Sheng Chen**: Resources. **Sharon Shui Yee Leung**: Resources, Writing – review and editing, Supervision, Project administration, Funding acquisition.

## Conflicts of Interest

The authors declare no conflicts of interest.

## Supporting information




**Supporting File**: advs75335‐sup‐0001‐SuppMat.docx

## Data Availability

The data that support the findings of this study are available from the corresponding author upon reasonable request.
