# Supplementary material for: A Chirality‐Converted Bacteriolytic Dodecapeptide Regulates Vibrio‐Induced Polymicrobial Infection and Ameliorates Invasion‐Associated Gut Microbiota Disequilibrium
Source: Adv Sci (Weinh). 2026 Apr 16;13(38):e22326. doi: 10.1002/advs.202522326 (PMC13335427; doi:10.1002/advs.202522326)
Supplement: Supplementary file 1 — Supporting File: advs75335‐sup‐0001‐SuppMat.docx [file ADVS-13-e22326-s001.docx]

**Supporting information**

**A chirality-converted bacteriolytic dodecapeptide regulates *Vibrio*-induced polymicrobial infection and ameliorates invasion-associated gut microbiota disequilibrium**

Ping Zeng ^a, #^, Qipeng Cheng ^b, c, #^, Xiaoxu Zhang ^a^, Honglan Wang ^a^, Jinghan Zhang ^b, c^, Xinyi Ding ^d^, Pengfei Zhang ^a^, Lanhua Yi ^e^, Kwok-Yin Wong ^f^, Kin-Fai Chan ^f^, Sheng Chen ^g, *^, Sharon Shui Yee Leung ^a, *^

^a^ School of Pharmacy, Faculty of Medicine, The Chinese University of Hong Kong, Shatin, Hong Kong;

^b^ Anhui Provincial Key Laboratory of Molecular Enzymology and Mechanism of Major Metabolic Diseases, College of Life Sciences, Anhui Normal University, Wuhu, Anhui, China;

^c^ Anhui Provincial Engineering Research Centre for Molecular Detection and Diagnostics, College of Life Sciences, Anhui Normal University, Wuhu, Anhui, China;

^d^ School of Pharmacy, Fudan University, Shanghai, China;

^e^ College of Food Science, Southwest University, Chongqing, China;

^f^ State Key Laboratory of Chemical Biology and Drug Discovery and Department of Applied Biology and Chemical Technology, The Hong Kong Polytechnic University, Hung Hom, Kowloon, Hong Kong;

^g^ Department of Food Science and Nutrition, Faculty of Science, The Hong Kong Polytechnic University, Hung Hom, Kowloon, Hong Kong.

**
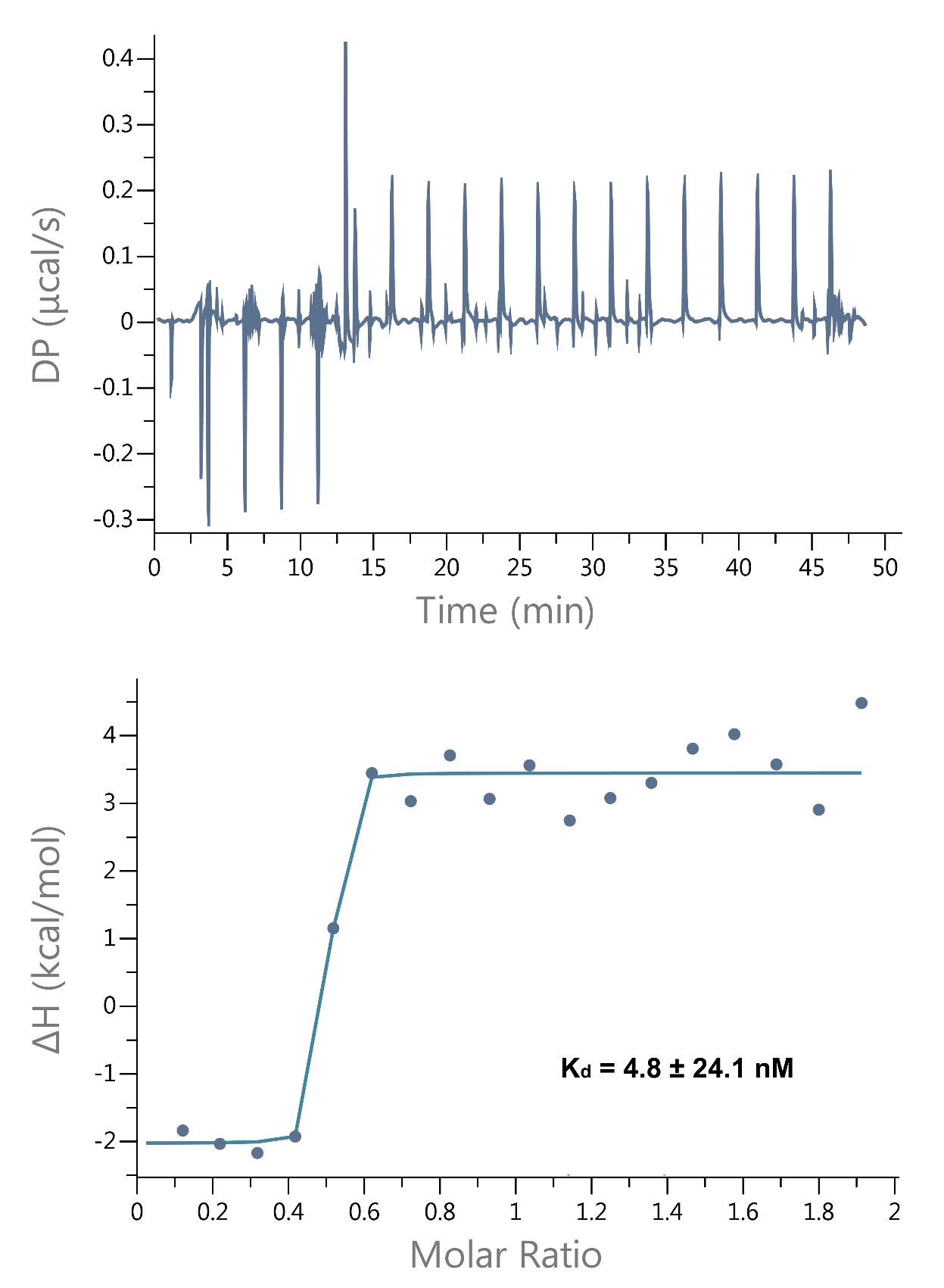
**

**Figure S1.** ITC result of **D-zp37** and LPS interaction.


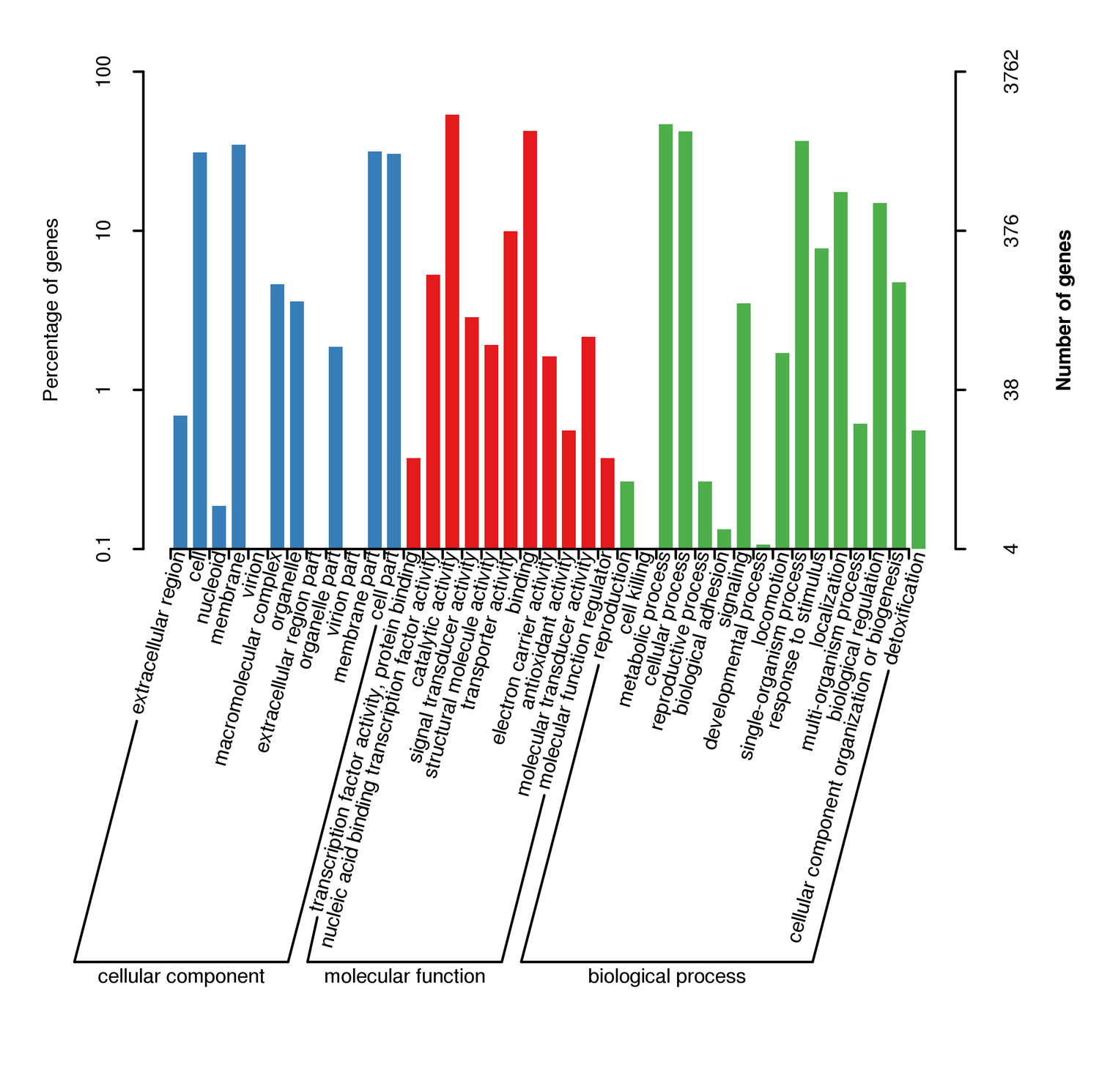


**Figure S2.** Classification statistics of gene ontology functional annotation of *VA* 1579.


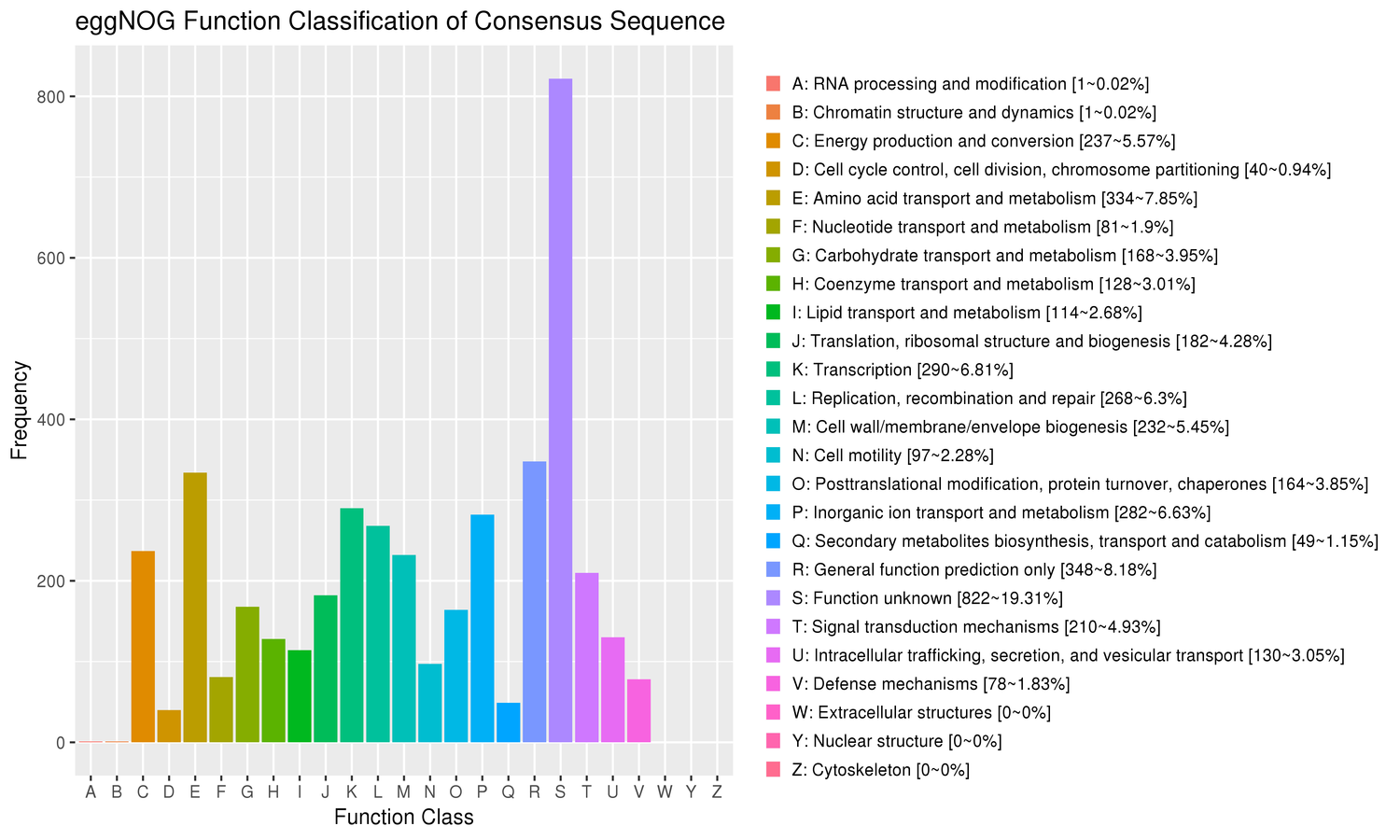


**Figure S3.** Classification statistics of eggNOG functional annotation of *VA* 1579.


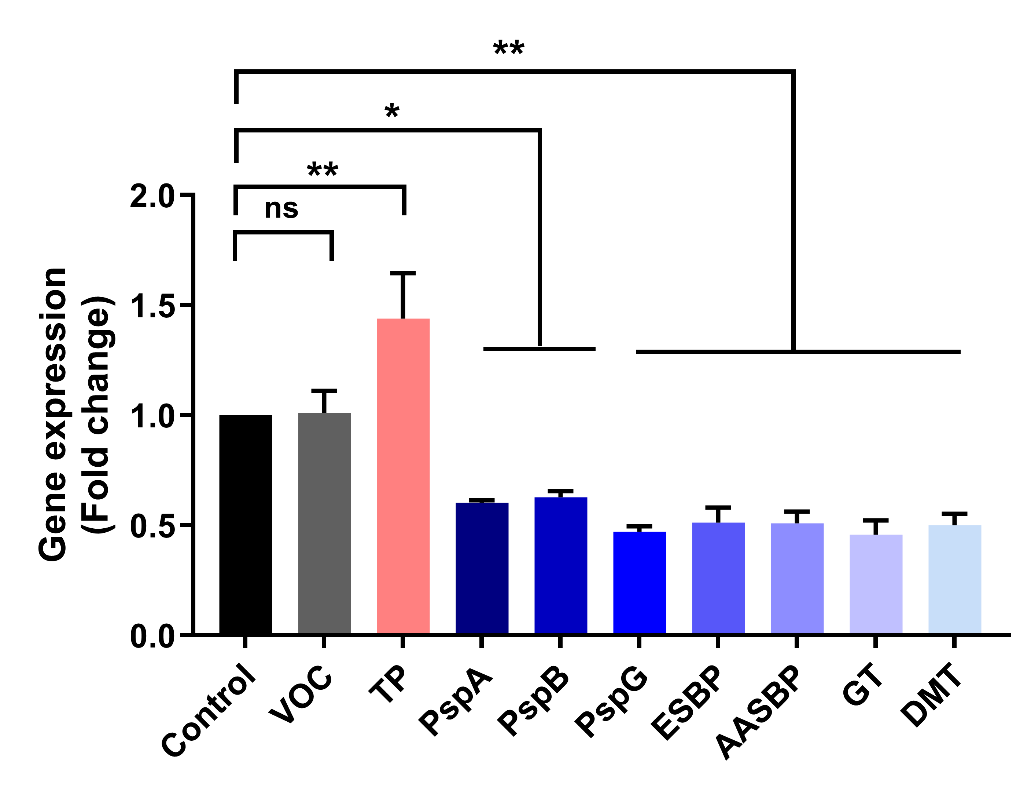


**Figure S4.** Real-time PCR analysis of nine selected genes. VOC, VOC family protein; TP, ABC transporter permease; Psp: phage shock protein; ESBP: extracellular solute-binding protein; AASBP: amino acid ABC transporter substrate binding protein; GT, glycosyltransferase family 2 protein; DMT, DMT family transporter.


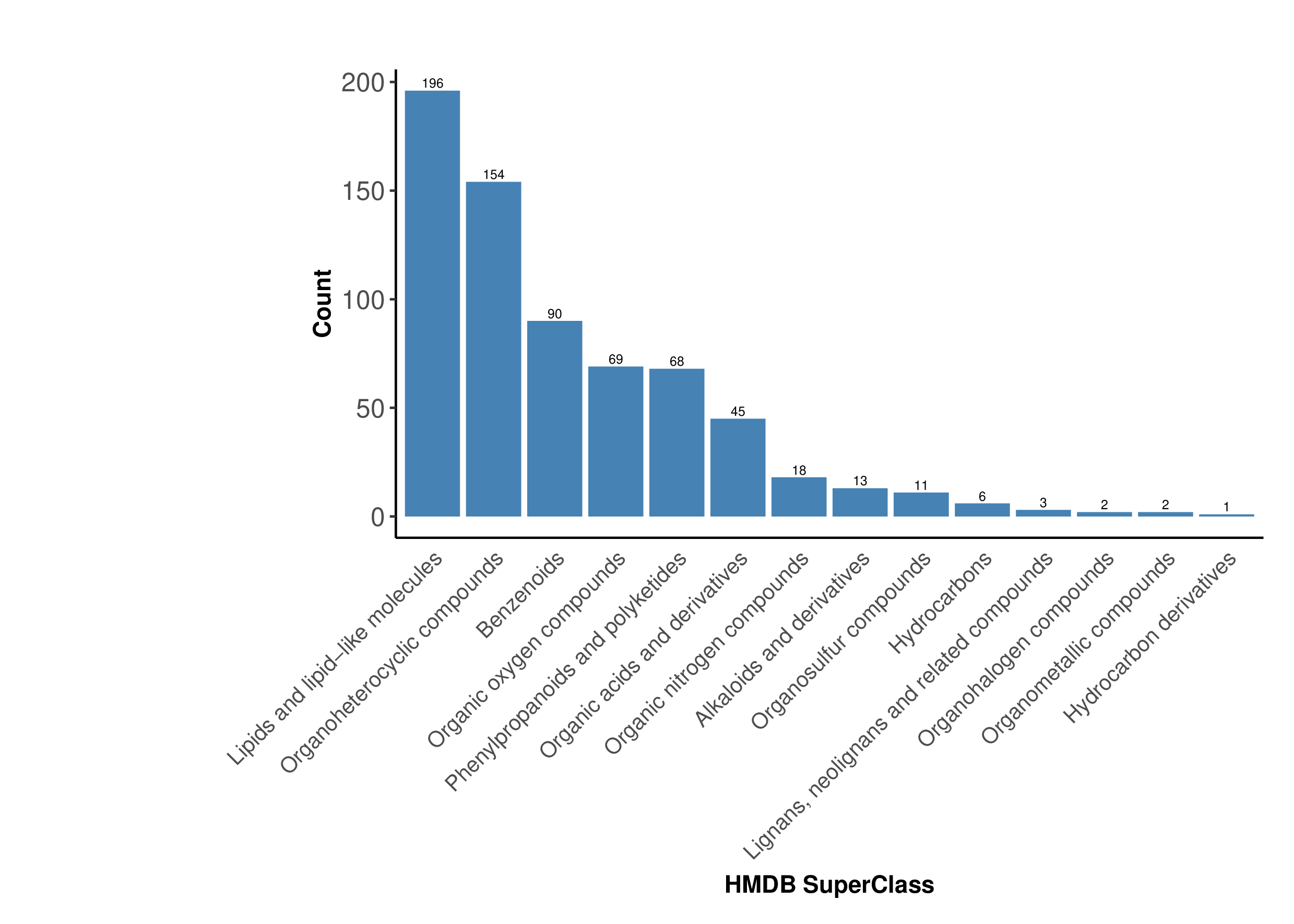


**Figure S5.** Human metabolome database annotation statistical chart of identified metabolites.

**
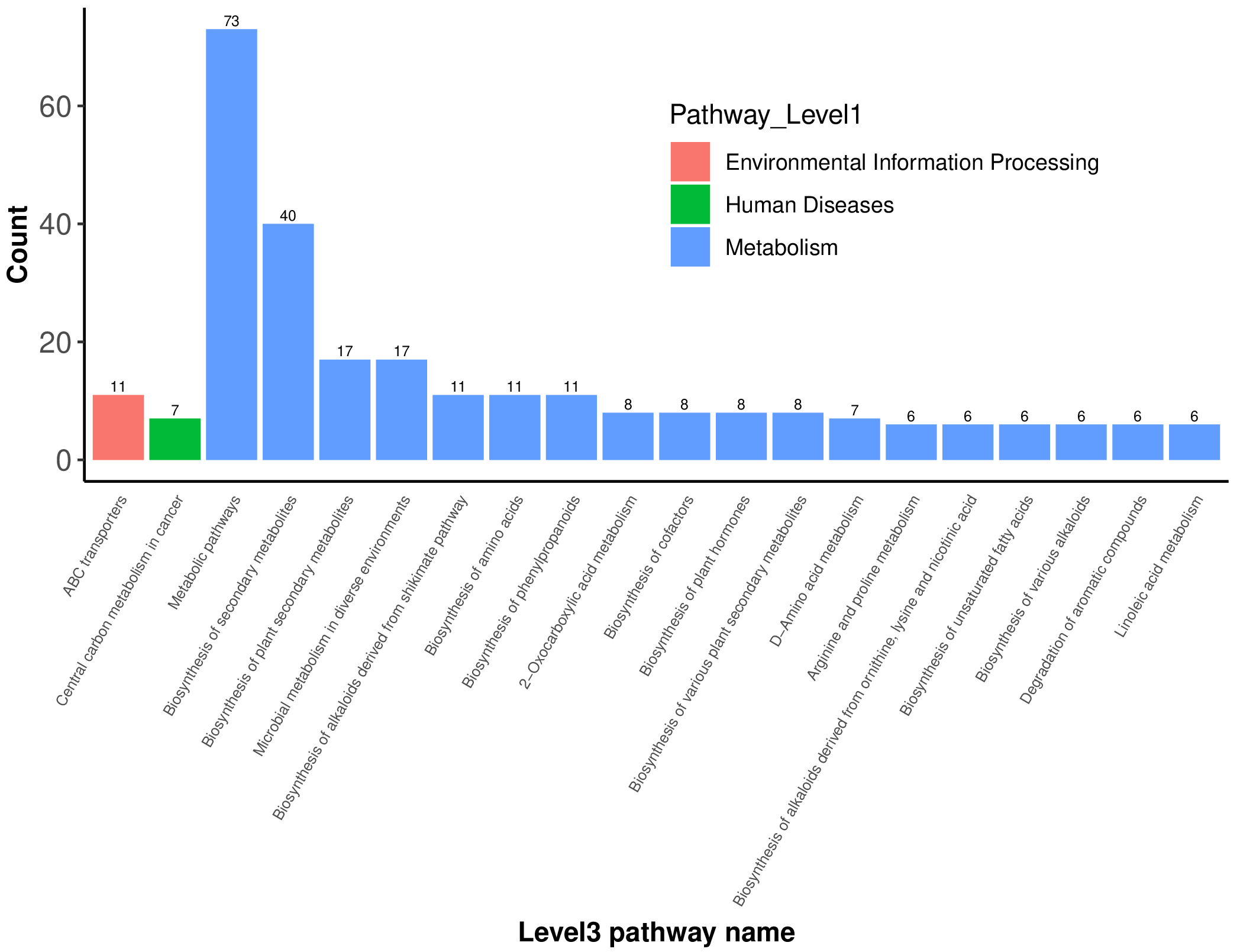
**

**Figure S6.** Top 20 pathways in terms of metabolite numbers based on KEGG database.


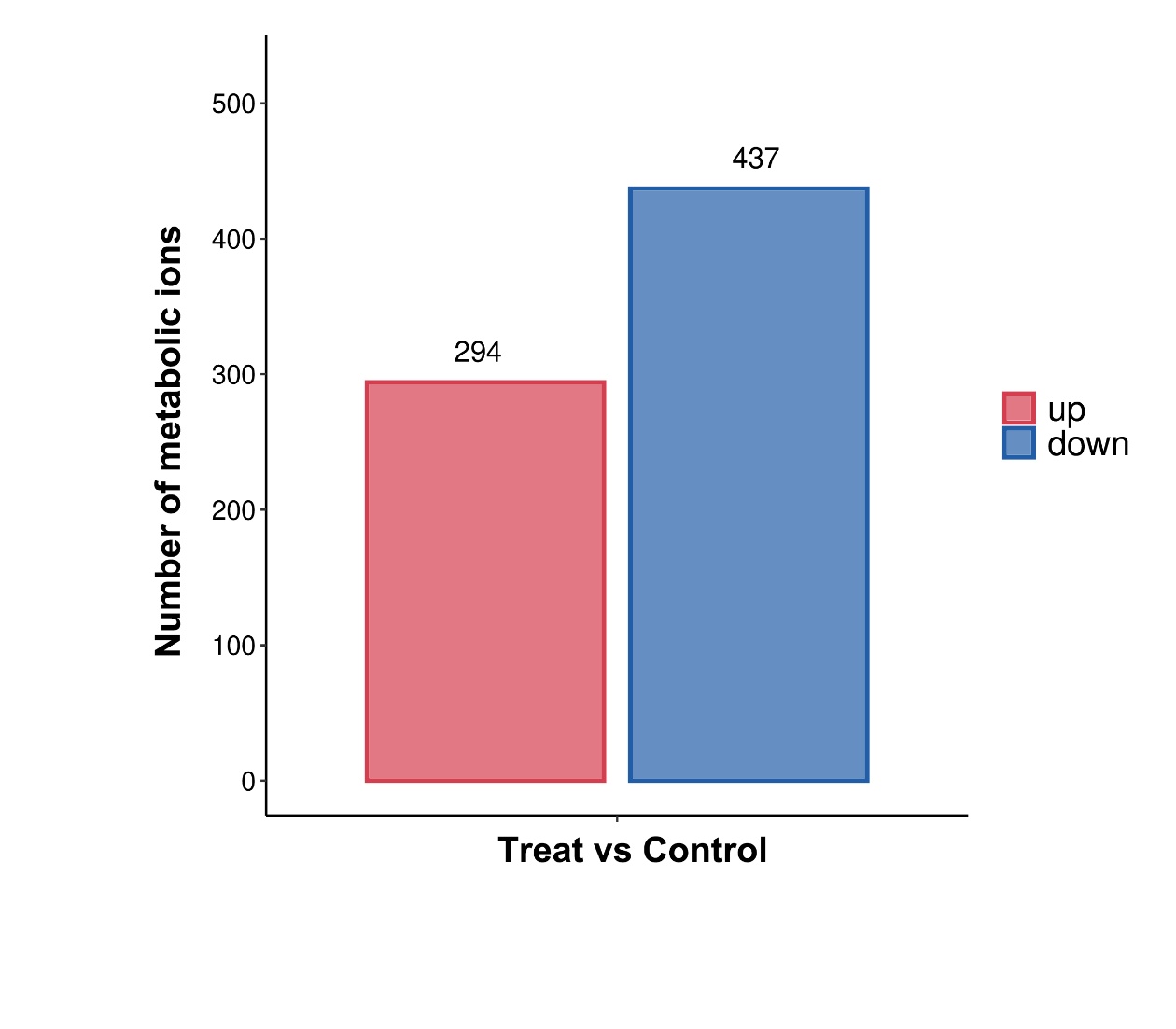


**Figure S7.** Number of metabolic ions before and after **D-zp37** treatment.


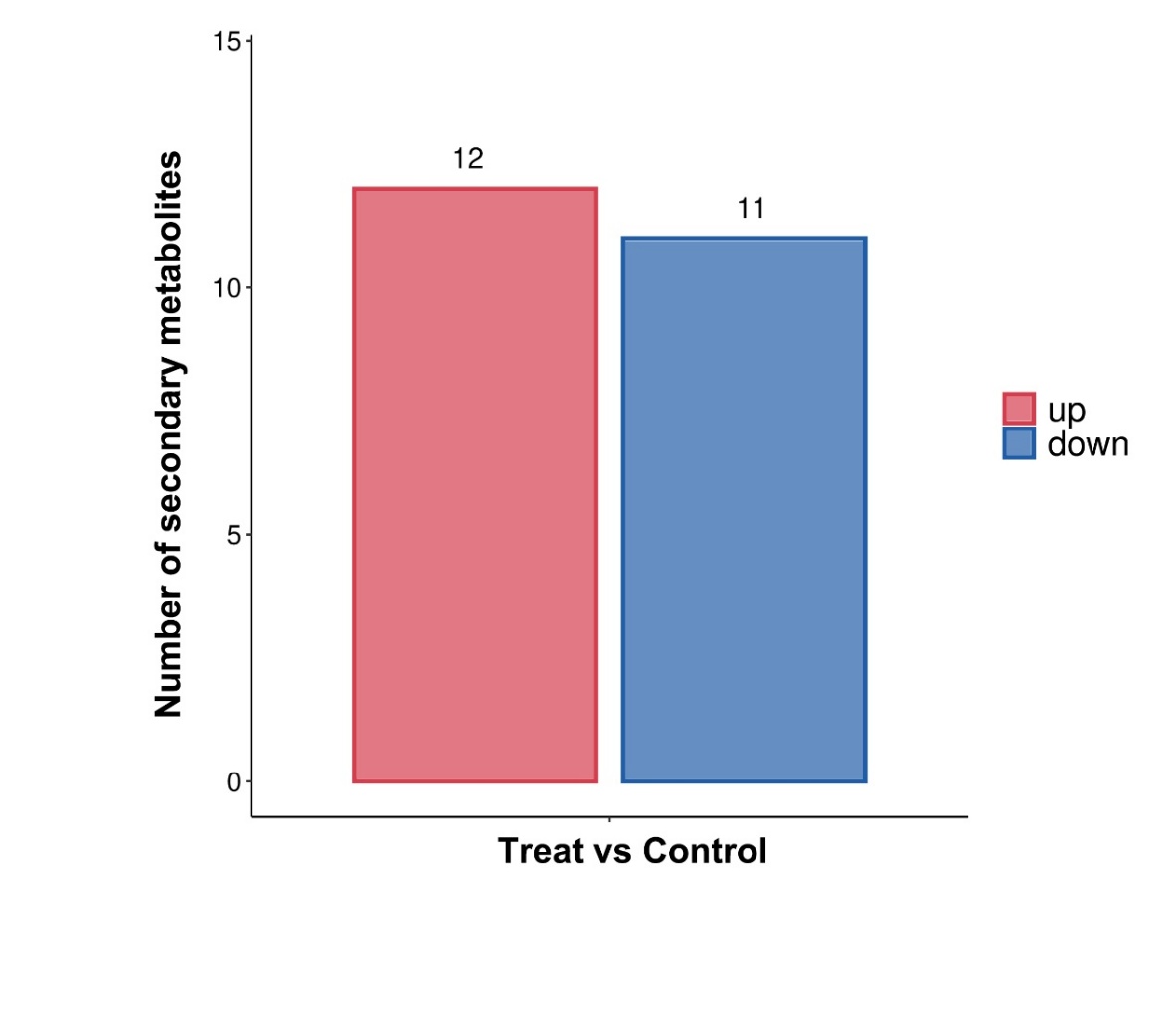


**Figure S8.** Number of secondary metabolites before and after **D-zp37** treatment.


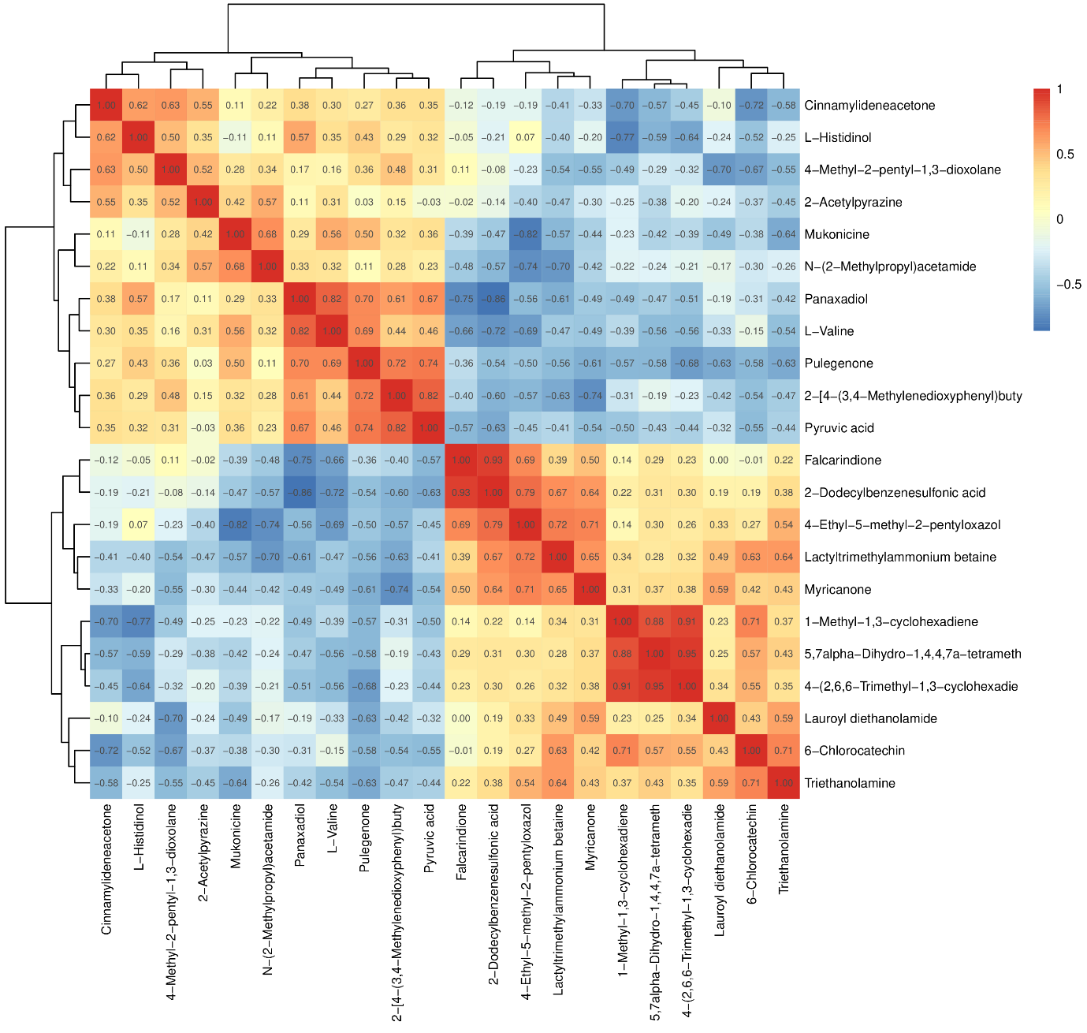


**Figure S9.** Correlation heat map of top 30 metabolites.

**
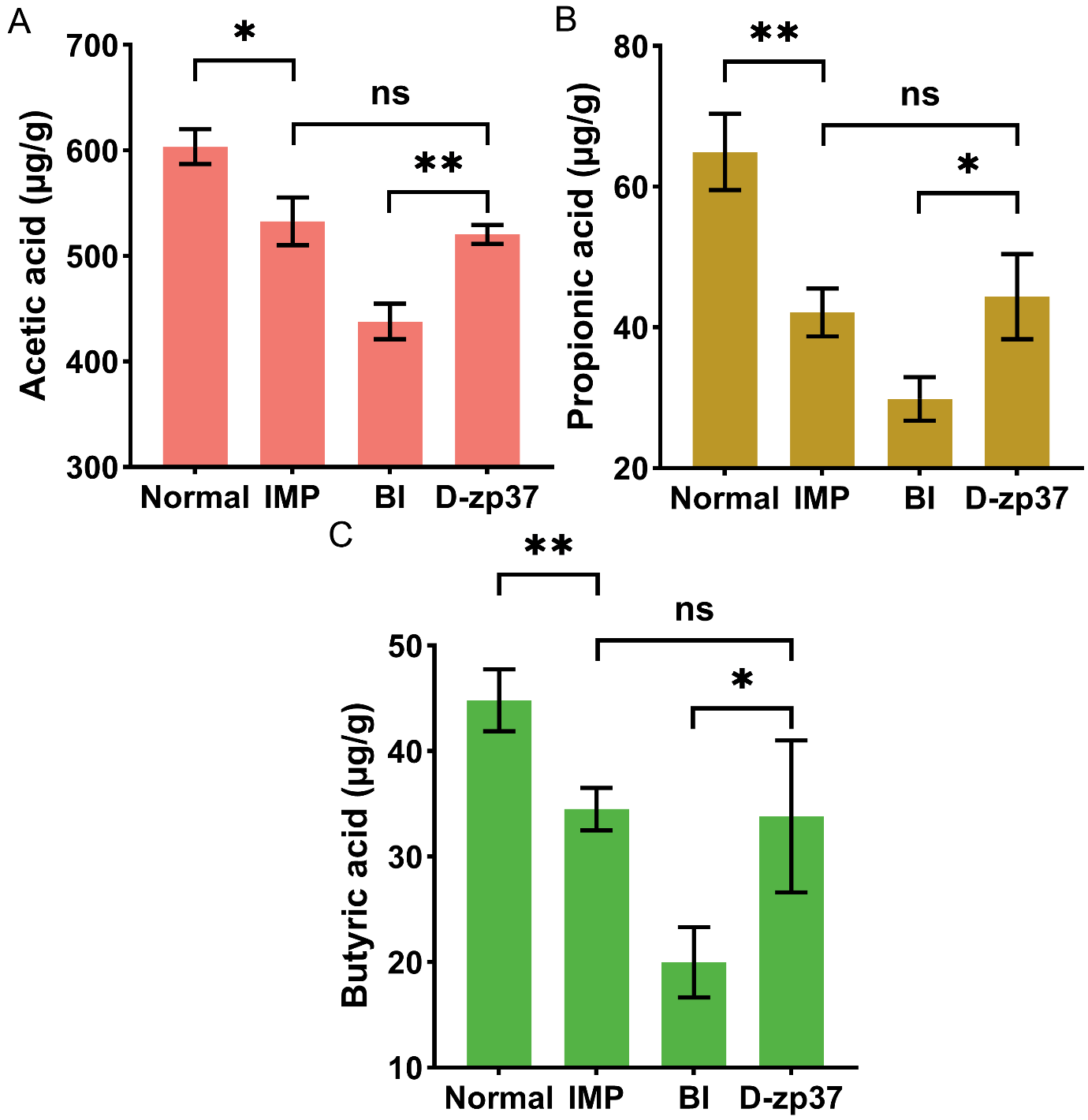
**

**Figure S10.** Comparison of fecal SCFA from mice with different treatment. (A) Acetic acid level. (B) Propionic acid level. (C) Butyric acid level.

**
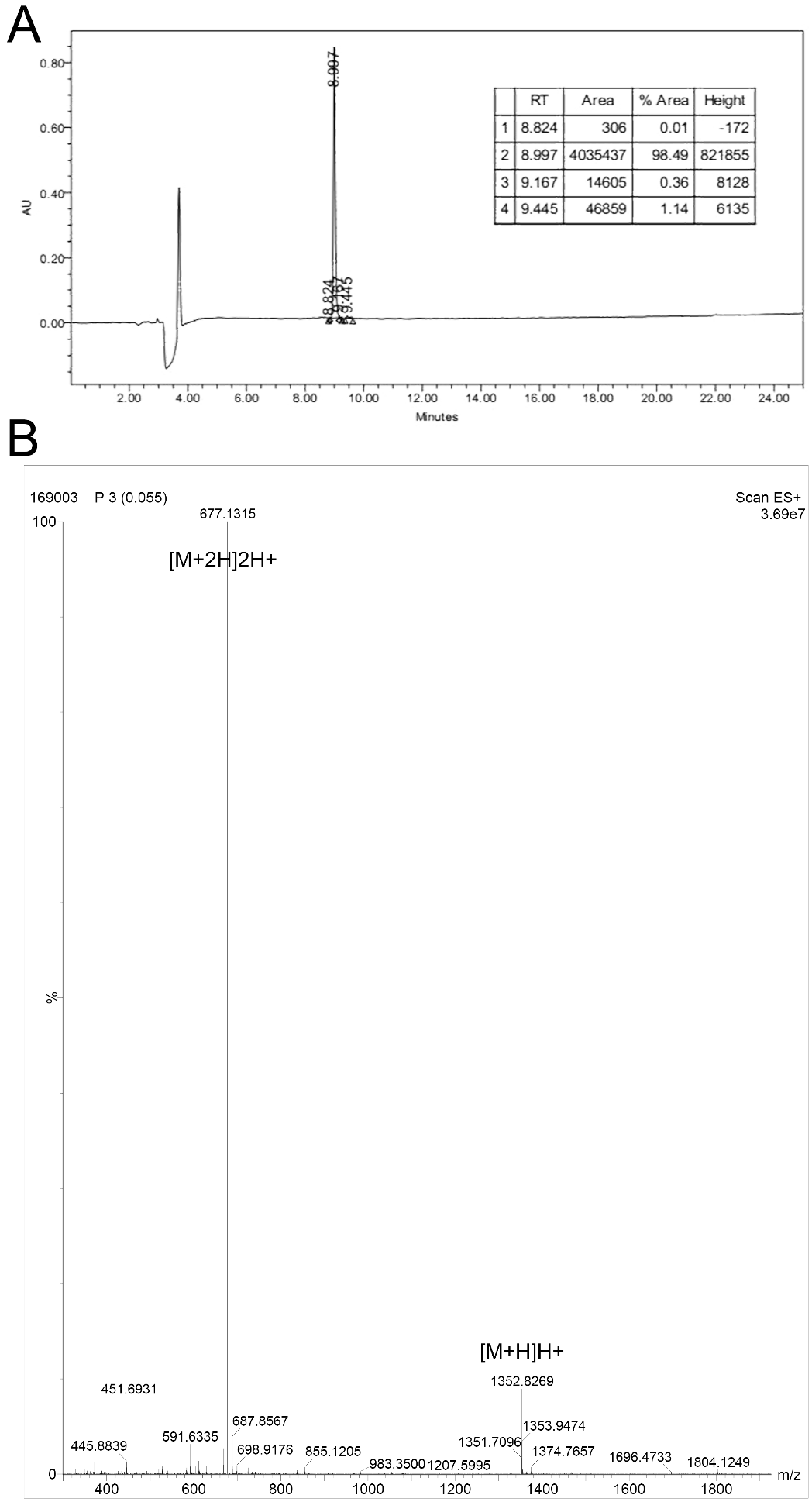
**

**Figure S11.** (A) HPLC spectra of purified **D-zp37**. (B) Electrospray-ionization mass spectra of purified **D-zp37**.


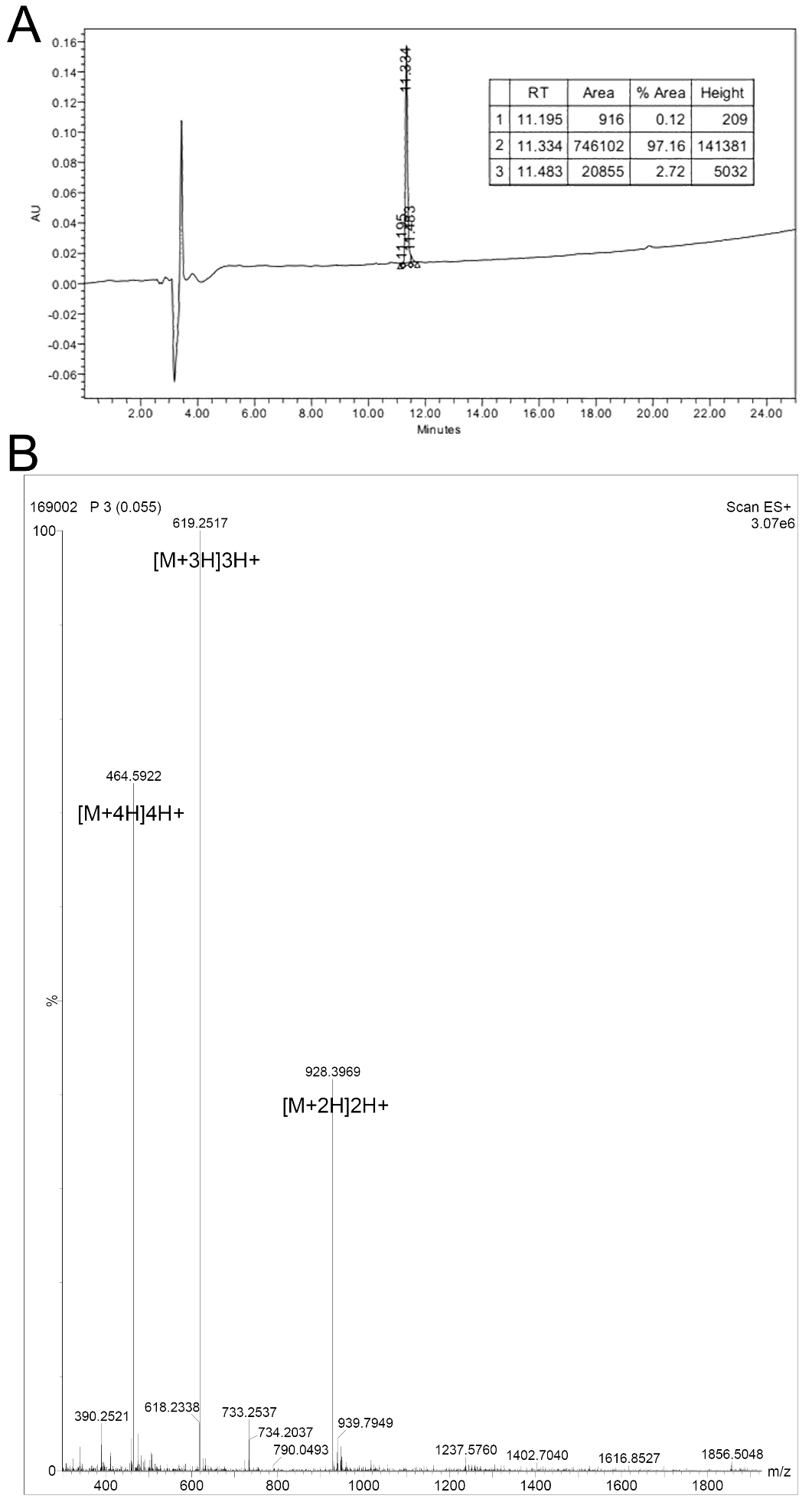


**Figure S12.** (A) HPLC spectra of purified **FITC-D-zp37**. (B) Electrospray-ionization mass spectra of purified **FITC-D-zp37**.

**Table S1.** Primers used in this study.

| **Primer name** | **Sequence (5’ to 3’)** |
| --- | --- |
| **Phage shock protein B-F** | TTTGTCGCTCCACTATGGCT |
| **Phage shock protein B-R** | ATCAACTCGGGACTGCATCG |
| **Phage shock protein A-F** | CCTCCACACTGAGCAAACCT |
| **Phage shock protein A-R** | TGCACATCACGACGGTTAGA |
| **Phage shock protein G-F** | GCAGCAACTGGTATTGCGTT |
| **Phage shock protein G-R** | ATTTATAGCGGGGCTGAGCG |
| **VOC family protein-F** | TGGCAAACCAAGAAACTGGC |
| **VOC family protein-R** | CATCGACGTTGTTGGTCACA |
| **DMT family transporter-F** | CAGCGATAATGGGCTTTGCC |
| **DMT family transporter-R** | TGGCAGTTTTCGAATGAGCA |
| **Extracellular SBP-F** | TCGCGATCCTGATATTGCCA |
| **Extracellular SBP-R** | TGCTTCGTTAAAGCCCCAGT |
| **Glycosyltransferase family 2 protein-F** | GGCGAAATTGGATGTGCCTG |
| **Glycosyltransferase family 2 protein-R** | CGTCACTTGCTTGAAACCCH |
| **ABC transporter permease-F** | TGTCATTCGCCTGCTGAACA |
| **ABC transporter permease-R** | AATCCCACAAACCCGTTGGA |
| **Amino acid ABC transporter SBP-F** | CAACCGAGCTAAACCTTGCAG |
| **Amino acid ABC transporter SBP-R** | AAGGCCAGATTGGTCAGTTGT |
